# TNF‐α‐Driven Changes in Polarized EGF Receptor Trafficking Facilitate Phosphatidylinositol 3‐Kinase/Protein Kinase B Signaling From the Apical Surface of MDCK Epithelial Cells

**DOI:** 10.1111/tra.70005

**Published:** 2025-05-05

**Authors:** Syntyche Ngalula, Cathleen R. Carlin

**Affiliations:** ^1^ Department of Molecular Biology and Microbiology, School of Medicine Case Western Reserve University Cleveland Ohio USA; ^2^ Case Western Reserve University Comprehensive Cancer Center, School of Medicine Case Western Reserve University Cleveland Ohio USA

**Keywords:** EGF receptor, epithelial polarity, inflammatory cytokines, PI3K/Akt signaling, unconventional protein secretion

## Abstract

This manuscript describes a novel unconventional secretory pathway that facilitates EGF receptor (EGFR) signaling from apical membranes in polarized epithelial cells responding to immune cell mediators. Epithelial tissues provide a physical barrier between our bodies and the external environment and share an intimate relationship with circulating and local immune cells. Our studies describe an unexpected connection between the proinflammatory cytokine tumor necrosis factor‐alpha (TNF‐α) and EGFR typically localized to basolateral membranes in polarized epithelial cells. These two molecules sit atop complex biological networks with a long history of shared investigative interest from the vantage point of signaling pathway interactions. We have discovered that TNF‐α alters the functional landscape of fully polarized epithelial cells by changing the speed and direction of EGFR secretion. Our results show apical EGFR delivery occurs within minutes of de novo synthesis likely via a direct route from the endoplasmic reticulum without passage through the Golgi complex. Additionally, our studies have revealed that apical cellular compartmentalization constitutes an important mechanism to specify EGFR signaling via phosphatidylinositol‐4,5‐bisphosphate 3‐kinase/protein‐kinase‐B pathways. Our study paves the way for a better understanding of how inflammatory cytokines fine‐tune local homeostatic and inflammatory responses by altering the spatial organization of epithelial cell signaling systems.

## Graphical abstract

Epithelial‐immune cell crosstalk is tightly regulated by secretion of immune cell mediators into luminal and serosal fluid spaces of epithelial tissues. We report two major new findings regarding how the inflammatory cytokine TNF‐α modifies epithelial cell function without impacting barrier function. TNF‐α activates an unconventional secretory pathway altering the polarized distribution of the EGF receptor and subsequently the spatial organization of epithelial cell signaling systems. Our studies have revealed epithelial cell biological mechanisms as unexpected targets for proinflammatory signaling.

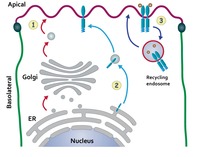

## 1 Introduction

Epithelial cell sheets and tubes are fundamental building blocks of most organs and tissues in all animal species [1, 2]. Cell polarity orchestrates the physical integrity of epithelia by determining the localization of specialized adhesion molecules mediating interactions between cells and with the underlying extracellular matrix and organizing the cytoskeleton and internal architecture of the cell. Epithelial functionality is also highly dependent on molecular mechanisms regulating targeted secretion of housekeeping and specialized cargoes to the apical (Ap) and basolateral (BL) membranes [3, 4, 5]. Biosynthetic secretory pathways involving coat protein complex II (COPII)‐dependent exit from the endoplasmic reticulum (ER), passage through the ER‐Golgi intermediate compartment (ERGIC) and Golgi stack, and exit from the trans‐Golgi network (TGN) in selectively targeted transport carriers have been intensely investigated for the past several decades [3, 4, 5]. Madin‐Darby canine kidney (MDCK) cells have provided a mainstay in vitro model for mammalian cells in many of these studies [6]. Classical biosynthetic sorting has turned out to be surprisingly complex, consisting of multiple seemingly redundant routes that often involve transit through endocytic compartments [3, 4, 5]. Unconventional sorting routes directing polarized cargo transport from the ER in non‐COPII vesicles or bypassing the Golgi altogether via mechanisms that are just coming into focus have recently been identified as well [7, 8, 9]. The existence of multiple independently regulated pathways provides balance between sorting fidelity and homeostatic flexibility, with potential links to epithelial pathologies through mis‐regulation.

Barrier epithelial tissues play crucial roles in immune defense by safeguarding the body's interior from a constant barrage of noxious environmental factors and pathogens [10]. Epithelia also have a central role in immune surveillance through a series of complex interactions with innate immune cells such as tissue‐resident macrophages and circulating leukocytes [11, 12, 13]. Innate immune cells release a variety of soluble factors that combat tissue injury and pathogen infection by initiating low‐grade inflammation followed by a resolution phase inducing immune tolerance and restoration of tissue homeostasis [14]. Epithelial‐immune cell crosstalk is tightly regulated by the secretion of immune cell mediators into luminal and serosal fluid spaces of epithelial tissues and the asymmetric distribution of cognate receptors on responding epithelial cells. Many immune cell factors are typically latent because ligands and receptors are strictly segregated unless the epithelial barrier is leaky. For instance, the critical pro‐inflammatory cytokine TNF‐α signals via one of its cognate receptors TNFR1 typically localized to Ap membranes in barrier epithelia [15, 16, 17]. Compartmentalized signaling enables TNF‐α secreted by circulating immune cells to act as an in vivo sensor of barrier function impairment [18, 19]. On the other hand, TNF‐α secreted by alveolar macrophages engages directly with the luminal surface of the alveolar space to help orchestrate a restorative inflammatory response during acute lung injury [20, 21]. However, the extent to which dynamic changes in cell polarity affect responses to inflammatory stimuli is largely unknown.

The epidermal growth factor receptor (EGFR) is a key player in the normal physiology of most epithelial tissues as well as numerous epithelial disorders with perturbed EGFR expression [22]. Increasing evidence suggests EGFR also has a major role balancing innate immune responses in multiple epithelial tissues [23, 24, 25]. EGFR possesses several sorting signals, including one recognizing the epithelial cell specific adaptor AP1B, directing its targeted delivery to BL membranes in most epithelial cells [26, 27, 28, 29]. However, EGFR is also present on Ap membranes during development and in some adult tissues suggesting a level of plasticity in its polar sorting itineraries [30, 31, 32, 33, 34, 35]. Additionally, its presence on Ap membranes indicates EGFR may have unique but poorly defined signaling capabilities from that compartment. EGFR signaling is primarily regulated by binding one of seven soluble ligands initially produced as membrane‐bound precursors in stromal fibroblasts as well as polarized epithelial cells where all but prototypic ligand EGF are delivered to BL membranes [36, 37]. Selective release of apically targeted EGF has an essential role in restoring barrier function after epithelial tissue injury [22]. It's been established that cell adhesion regulates EGFR signaling by restricting ligand‐induced trafficking and signaling on BL membranes [38]. However not much else is known about how cell polarity impacts EGFR signaling particularly recruitment of downstream EGFR signaling molecules. Recent studies have also identified a new epithelial membrane compartment under control of the polarity regulatory Crumbs complex located on the Ap side of the tight junctional adhesion barrier [39]. Already linked to the Hippo pathway and cytoskeletal regulation, the potential involvement of this “vertebrate marginal zone” in other signaling pathways is an exciting new area of investigation for epithelial cell signaling compartmentalization [39].

Prior studies have shown that activation of chemokine receptors on the Ap membrane of epithelial cells can alter host‐pathogen interactions by modulating the polarized distribution of select membrane protein cargo [40, 41]. Since EGFR signaling networks are common targets during host responses to microbial pathogens [42], these findings piqued our interest regarding whether EGFR behavior might be similarly regulated during immune sensing. We have tested this hypothesis in the MDCK cell model, demonstrating that long‐term exposure to luminal TNF‐α induced EGFR sorting to Ap membranes in preformed monolayers via an unconventional secretory pathway. This change in EGFR polarity was associated with a significant increase in ligand‐induced EGFR signaling through the phosphoinositide 3‐kinase/protein kinase B (PI3K/Akt) pathway, with little impact on other EGFR responses from Ap membranes. These studies have revealed an unexpected role for TNF‐α in organizing polarized EGFR responses to inflammatory stimuli.

## 2 Results

### 2.1 Modeling Compartmentalized Innate Immune Signaling in Polarized MDCK Cells

**FIGURE 1:**
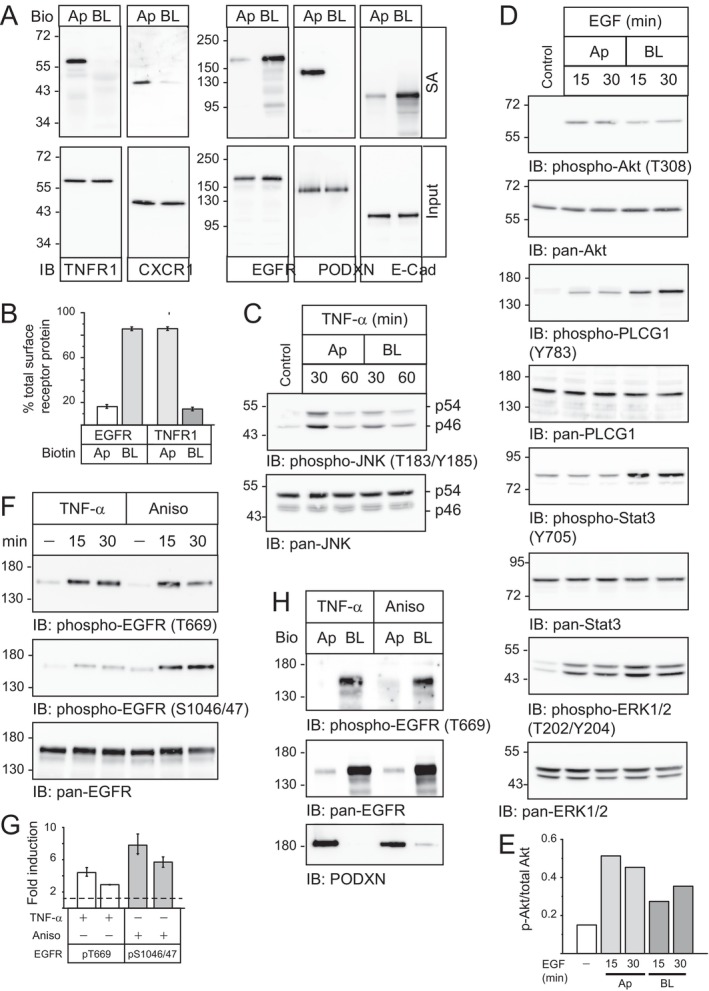
MDCK cells as an in vitro model of compartmentalized signaling in polarized epithelia. (A) Filter grown MDCK‐II cells with stable CMV‐driven human EGFR expression (WT‐EGFR) were labeled with a membrane impermeant biotinylating reagent added to Ap and BL membrane surfaces. Streptavidin (SA)‐purified proteins and equal aliquots of input lysates (25 μg) were immunoblotted with antibodies to TNF‐α and IL‐8 receptors (TNFR1 and CXCR1 respectively) (left panels), and EGFR and resident Ap (PODXN) and BL (E‐Cad) (right panels) membrane proteins. (B) EGFR and TNFR1 protein bands in (A) were normalized to their input controls, and data were presented as percentage domain‐specific expression relative to total membrane‐exposed protein (mean ± SEM, *n* = 3). (C, D) Filter‐grown monolayers were stimulated with TNF‐α (C) and EGF (D) (10 ng/mL each) to characterize acute domain‐specific responses by immunoblot analysis of equal aliquots of total cellular protein (25 μg) with phospho‐specific and pan antibodies to signaling molecules listed in the figure. (E) Phospho‐specific and total Akt protein bands in (D) were quantified and data were presented as the ratio of Thr308 phosphorylation relative to total Akt protein. (F) Filter‐grown WT‐EGFR cells were harvested to determine EGFR responses to acute stimulation with TNF‐α (10 ng/mL) and anisomycin (25 μg/mL) by immunoblot analysis with phospho‐specific EGFR antibodies recognizing MAPK substrates (Thr669 and Ser1046/1047) and a pan‐EGFR antibody to control for protein loading. (G) Phospho‐specific EGFR bands in (F) were normalized to their input controls and data were presented as fold induction relative to unstimulated cells (represented by the dashed line) (mean ± SEM, *n* = 3). (H) Filter‐grown cells were biotinylated at Ap and BL membrane surfaces prior to an acute 30‐min stimulation with TNF‐α and anisomycin. Cells were harvested for recovery of biotin‐labeled proteins by SA purification and immunoblotting with antibodies to EGFR [phospho‐specific (Thr669) and pan] and PODXN to control for Ap domain biotinylation.

We first established that MDCK cells were a suitable in vitro model for examining innate immune sensing by epithelial barriers. Mature MDCK monolayers were prepared using established laboratory protocols [26, 27, 28, 29]. Briefly, actively growing cells were seeded on permeable polycarbonate filter supports (0.4 μm pore size) at high density (2.0 × 10^5^ cells/cm^2^) to saturate the surface area. We have shown previously that high density monolayers achieved steady state transepithelial electrical resistance (TEER) values of approximately 160 Ohms/cm^2^ after 24 h and were functionally and morphologically polarized with homeostatic cell numbers when studies were carried out between 4 and 6 days of seeding [26, 27, 28, 29]. Membrane‐bound receptors for the pro‐inflammatory cytokines TNF‐α and IL‐8 [Tumor necrosis factor receptor 1 (TNFR1) and CXC chemokine receptor 1 (CXCR1) respectively] were primarily localized to Ap membranes based on their streptavidin (SA) affinity purification from filter‐grown cells labeled with a membrane impermeant biotin reagent added to Ap and BL cell surfaces (Figure 1A, left panels; Figure 1B). We have shown previously that approximately 90% of total surface EGFRs were localized on BL membranes in a human EGFR overexpression MDCK cell model [28, 29, 43]. Results in Figure 1A (right panels) and Figure 1B agreed with those findings (Figure S1). Domain‐specificity of biotin labeling was verified by examining polarized distributions of resident Ap and BL membrane proteins podocalyxin (PODXN) and E‐cadherin (E‐Cad) respectively (Figure 1A, right panels). The biotin labeling results were extended by immunoblotting whole cell lysates with phosphorylation‐specific antibodies recognizing activated forms of various signaling molecules shortly after receptor ligands were added to Ap and BL sides of the cell. c‐Jun N‐terminal kinase (JNK) isoforms were activated within 30 min of TNF‐α addition to the Ap compartment (Figure 1C). Multiple EGFR‐mediated signaling responses were also analyzed. EGFRs exhibited a relatively poor PI3K/Akt response to acute EGF stimulation from either membrane domain (Figure 1D,E). Since EGFR utilizes indirect mechanisms for activating this pathway [44], this result suggested distributions of intermediary molecules linking EGFR to PI3K/Akt were tightly regulated by cell polarity. However, two other signaling pathways involving phospholipase Cγ1 (PLCG1) and signal transducer and activator of transcription 3 (Stat3) exhibited expected selective responses to acute stimulation with BL ligand (Figure 1D). In contrast, a relatively small percentage of EGFRs present on Ap membranes appeared to be sufficient to induce significant activation of the ERK1/2 mitogen‐activated protein kinases (MAPKs) following EGF stimulation (Figure 1D). Altogether these findings underscored the importance of examining specific signaling pathways when considering effects of EGFR polarity on downstream targets. EGFR is also known to undergo p38‐MAPK‐dependent phosphorylation downstream of several cell stress inducing agents including TNF‐α independent of canonical EGF ligands [45]. We therefore asked whether TNFR1 and EGFR localization to opposing membrane domains affected these responses. EGFR phosphorylation at two p38‐MAPK‐dependent substrates (Thr669 and Ser1046/1047) was examined with phospho‐specific EGFR antibodies following acute stimulation with TNF‐α as well as the p38‐MAPK inducing antibiotic anisomycin [46]. Although EGFR phosphorylation was induced by both stimuli, anisomycin triggered a stronger response particularly at the Ser1046/1047 EGFR substrate compared to TNF‐α (Figures 1F,G and S1). Not surprisingly, both stress‐inducing agents specifically targeted BL EGFRs for Thr669 phosphorylation within minutes of stimulation based on a biotin labeling assay (Figure 1H). These results suggested luminal TNF‐α was capable of transmitting signals across MDCK barriers via EGFR crosstalk by a mechanism that was both rapid and unimpeded by epithelial cell architecture.

### 2.2 Long‐Term TNF‐α Exposure Promoted Enhanced EGFR Expression on the Ap Cell Surface of Preformed MDCK Monolayers

**FIGURE 2:**
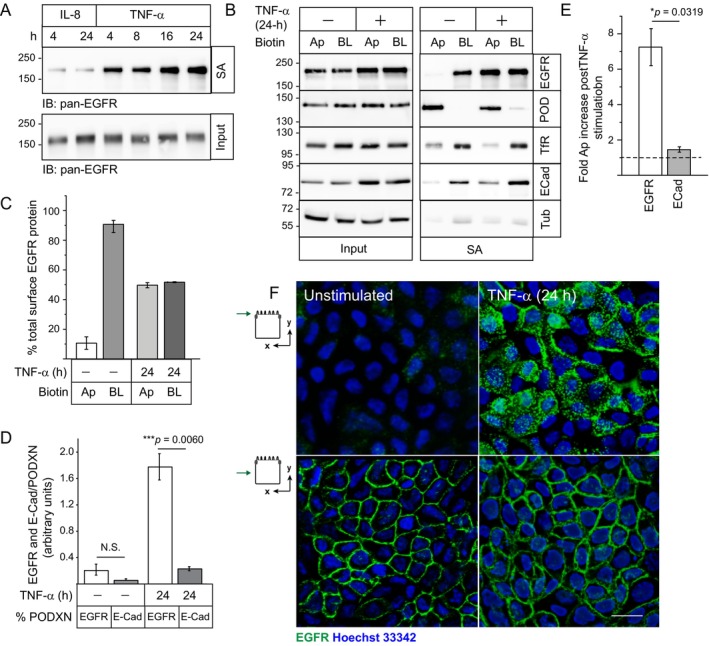
Long term TNF‐α treatment altered the spatial distribution of EGFR across a polarized MDCK epithelial cell model. (A) Filter‐grown cells were subjected to long term IL‐8 and TNF‐α exposure (10 ng/mL each) for times indicated followed by biotin labeling of Ap cell surface‐exposed proteins and cell harvesting for immunoblot analysis of SA‐purified and input aliquots (25 μg) with a pan‐EGFR antibody. (B) Filter‐grown cells were biotinylated at both membrane surfaces without additional treatment (−) and following long term TNF‐α (10 ng/mL) stimulation (+) for 24 h for immunoblot analysis of input lysates (25 μg) and SA‐purified proteins using antibodies listed in the figure. (C) EGFR protein bands in (B) were normalized to their tubulin input controls, and data presented as percentage domain‐specific expression relative to total membrane‐exposed protein (mean ± SEM, *n* = 3). (D) Apically biotinylated PODXN, EGFR, and E‐Cad protein bands in (B) were normalized to their input controls, and data presented as percentage EGFR (white bars) and E‐Cad (grey bars) expression relative to PODXN under unstimulated (−) and TNF‐α‐treated (+) conditions (mean ± SEM, *n* = 3). ***p = 0.0060 for TNF‐α‐treated cells (Student's *t* test). (E) Ap EGFR and E‐Cad protein bands in (B) were normalized to their input controls and data presented as fold increase in Ap EGFR and E‐Cad expression following a 24‐h TNF‐α treatment compared to untreated cells (represented by dashed line). *p = 0.0319 for Ap EGFR expression relative to Ap E‐Cad (Student's *t* test) (mean ± SEM, *n* = 3). (F) Representative two‐dimensional *X*–*Y* optical images (~ 0.5 mm thickness) of non‐permeabilized filter‐grown MDCK monolayers stained with an EGFR antibody recognizing a surface exposed epitope (green) added to Ap and BL surfaces and counterstained with Hoechst 33342 (blue) to visualize nuclei under unstimulated and TNF‐α‐treated conditions. Icons indicate relative position of Z‐stack image. Size bar, 5 μm.

**FIGURE 3:**
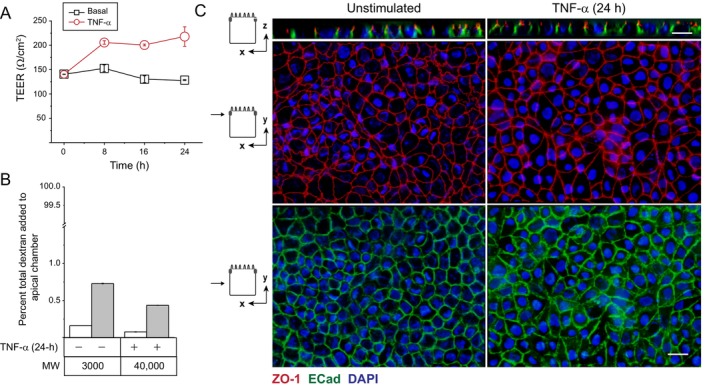
Long term TNF‐α treatment did not have a significant impact on epithelial barrier function. (A and B) The effect of long‐term TNF‐α exposure (10 ng/mL) on barrier integrity was determined by measuring TEER expressed as Ω/cm^2^ at times indicated (A); and Ap‐to‐BL flux of fluorescently labeled dextran tracers of various size (3000 and 40 000 MW) expressed as percentage of total tracer added to the Ap chamber over a 30‐min period at the 24‐h post‐stimulation time point (B). Data presented as mean ± SEM (*n* = 3). (C) Representative two‐dimensional *X*–*Z* and *X*–*Y* optical images (~ 0.5 μm thickness) of filter‐grown MDCK monolayers stained with antibodies to ZO‐1 (red) and E‐Cad (green) and counterstained with DAPI (blue) to visualize nuclei under unstimulated and TNF‐α‐treated conditions. Size bars, 5 μm.

Fully polarized monolayers were exposed to physiological concentrations of luminal IL‐8 and TNF‐α, and Ap EGFR expression was examined by domain‐specific biotin labeling assay from 4 to 24 h later. Although IL‐8 had a negligible effect, TNF‐α exposure was associated with increasing Ap EGFR expression commencing at 4 h and continuing to 24 h post stimulation (Figure 2A). The TNF‐α‐mediated response was extended by comparing Ap and BL membrane domain distributions of EGFR as well as resident Ap (PODXN) and BL (E‐Cad and TfR) membrane proteins at the 24‐h post stimulation time point (Figure 2B). Domain‐specific biotin labeling assays revealed that EGFRs exhibited non‐polar expression with quantitatively similar abundance on both membrane domains (Figures 2B,C and S2). We also quantified Ap expression of EGFR and the BL marker E‐Cad relative to the resident Ap membrane protein PODXN (EGFR/PODXN and E‐Cad/PODXN respectively) under different experimental conditions. TNF‐α exposure was associated with a statistically significant increase in EGFR/PODXN while having a negligible effect on E‐Cad/PODXN (Figures 2D and S2). Additionally, quantitative analysis revealed that long‐term TNF‐α treatment was associated with an approximately seven‐fold induction in Ap EGFR expression with negligible effect on Ap E‐Cad expression (Figure 2E). Collectively, these data ruled out a global TNF‐α‐regulated effect on polarized trafficking. Biochemical biotin labeling assays were confirmed by staining non‐permeabilized cells with EGFR1 monoclonal antibody recognizing a surface‐exposed EGFR epitope added to Ap and BL surfaces of filter‐grown cells. Images from cells stained from Ap membranes revealed that TNF‐α‐treated cells displayed a punctate EGFR expression pattern characteristic of Ap microvillar staining in Z‐stack images taken in the plane of the cell apex compared to untreated cells with negligible EGFR staining (Figure 2F, upper panels; Figure S3). In contrast, cells stained from BL membranes had similar EGFR distributions with a characteristic cobblestone appearance in Z‐stack images from the mid‐point of the BL domain under both conditions (Figure 2F, lower panels; Figure S3). Permeabilities of ionic and nonionic molecules were also evaluated by measuring transepithelial electrical resistance (TEER) (Figure 3A) and Ap‐to‐BL paracellular permeability of different sized dextran fluorescent tracers (3000 vs. 40 000 MW) over a 30‐min interval at 37°C (Figure 3B) respectively. Not unexpectedly, since these two parameters are independently regulated [47], TEER and dextran flux were both increased in TNF‐α‐treated cells. Mature monolayers of MDCK II cells used in this study typically display relatively low TEERs between 100 and 300 Ω/cm^2^ [48], and the increase seen in TNF‐α‐treated cells fell within this range (Figure 3A). Although there were slight size‐dependent increases in dextran flux, these measurements were still less than 1% of the initial amount of dextran added to the Ap compartment (Figure 3B). Finally, we observed that MDCK cells maintained normal epithelial cell morphology based on examination of images obtained from cells co‐stained for E‐Cad and the tight junction protein ZO‐1 (Figure 3C). Altogether, these results supported a working model that our experimental conditions (10 ng/mL TNF‐α for 24 h) promoted enhanced EGFR expression on Ap membranes without a significant impact on epithelial cell architecture or a paracellular leakage pathway attributed to approximately ten‐fold higher concentrations of TNF‐α in some epithelial cell models [49, 50].

### 2.3 Long‐Term TNF‐α Exposure Promoted Ap Delivery of de Novo Synthesized EGFR via an Unconventional Secretory Pathway

**FIGURE 4:**
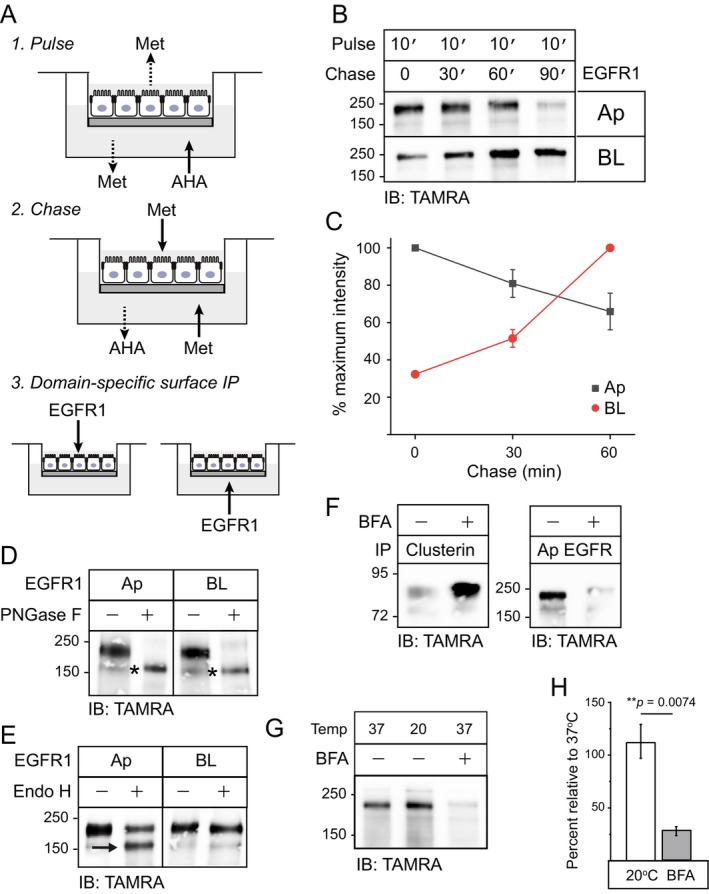
Long term TNF‐α exposure induced unconventional EGFR secretion to Ap membranes. (A) Schematic showing experimental strategy for capturing domain‐specific delivery of newly synthesized EGFR using copper‐free click chemistry. Briefly, confluent monolayers of filter‐grown cells were methionine (Met)‐starved for 30 min followed by a 10‐min incubation with Met‐free media supplemented with 50 mM of the azide‐modified Met analog AHA added to the BL surface (*Pulse*). Filters were rinsed 2 times and refed with Met‐supplemented complete media for various times (*Chase*), followed by a 1‐h incubation on ice with EGFR1 antibody added to either Ap or BL surfaces (*Domain‐specific surface IP*). Cell surface immunoprecipitants were subsequently labeled with an azide‐reactive TAMRA‐DBCO probe to facilitate detection of metabolically labeled EGFRs by immunoblotting with TAMRA antibody after SDS‐PAGE. (B) Representative immunoblots showing domain‐specific delivery of newly synthesized EGFRs following a long‐term (20‐h) TNF‐α exposure based on protocol in (A). (C) EGFR protein bands in (B) were quantified and data presented as the percent of EGFR band with most intensity which was set to 100 at time 0 for Ap EGFR (black line and symbols) and 60‐min for BL EGFR (red line and symbols) (mean ± SEM, *n* = 3). (D and E) TNF‐α treated (22 h) cells were pulse‐labeled for 10 min followed by 30‐ and 90‐min chase periods for recovery of EGFRs delivered to Ap and BL domains respectively. TAMRA‐DBCO reacted EGFR1 immunoprecipitants were mock‐treated or digested with PNGase F (D) and EndoH (E) for immunoblot analysis with TAMRA antibody. Asterisks and arrow denote migration of EGFR PNGase F (D) and EndoH (E) digestion products respectively. (F) Filter‐grown TNF‐α‐treated WT‐EGFR cells were pulse (10‐min)/chase (1 h) labeled in the absence or presence of BFA (1 μg/mL). Newly synthesized molecules were detected by TAMRA immunoblot analysis of clusterin (left panel) and EGFR (right panel) immunoprecipitants recovered from culture supernatant collected from the Ap chamber and the Ap cell surface and respectively. (G) Filter‐grown TNF‐α‐treated WT‐EGFR cells were pulse (10‐min)/chase (1 h) labeled at 37°C and 20°C (left and middle lanes), and at 37°C in the presence of BFA (1 μg/mL) (right lane). Delivery of newly synthesized EGFRs to the Ap cell surface was detected by TAMRA immunoblot analysis of Ap domain specific EGFR1 immunoprecipitants. (H) EGFR protein bands in (G) were quantified and data presented as percent Ap recovery collected from cells incubated at 20°C (white bars) and with BFA (grey bars) relative to cells incubated at 37°C (mean ± SEM, *n* = 3). **p = 0.0074 (Student's *t* test).

We next asked whether TNF‐α exposure affected polarized EGFR trafficking by adapting click chemistry to capture domain‐specific delivery of newly synthesized receptors (Figure 4A) [51]. Briefly, filter‐grown cells were methionine‐starved, pulse‐labeled with the azide‐modified methionine analog L‐azidohomoalanine (AHA), and washed and re‐incubated with methionine‐supplemented media for various intervals. Cells were then placed on ice and incubated with EGFR1 antibody added to either the Ap or BL side of the filter. Cell surface EGFR1 immunoprecipitants were isolated from whole cell lysates by affinity purification on protein G Sepharose beads, labeled with an azide‐reactive TAMRA‐DBCO probe, resolved by SDS‐PAGE, and immunodetected with TAMRA‐specific antibody. Although this assay revealed that newly synthesized receptors were detected on both membrane domains in TNF‐α‐treated cells there were notable differences in delivery kinetics. Consistent with studies carried in cells without long‐term TNF‐α treatment (Figure S5) and our previous results obtained by conventional pulse‐chase labeling with radioactive amino acids [26, 28], EGFRs exhibited maximal accumulation on BL membranes by the 60‐min chase time point (Figure 4B,C). In contrast, apically delivered EGFRs were detected during the pulse label with modest reduction in signal intensity by dilution with unlabeled receptors during a 60 min methionine chase (Figures 4B,C and S4). These studies were extended with a series of experiments based on recent evidence that unconventional Golgi bypass pathways tend to involve a faster process than canonical trafficking through the Golgi [7]. We first determined the types of N‐glycans attached to EGFR delivered to different membrane domains. It is well‐established that EGFR is initially modified by co‐translational addition of 7 to 9 high mannose N‐linked glycans in the ER that are subsequently remodeled forming complex N‐glycans by Golgi‐resident enzymes on route to plasma membrane [52, 53]. N‐glycan remodeling changes sensitivity to the enzyme endoglycosidase H (EndoH) that removes high mannose N‐glycan cores but not complex oligosaccharides from glycoproteins [54, 55], providing a convenient way for determining whether EGFR trafficked through the Golgi before appearing on different membrane domains. Nascent EGFRs on both membrane domains displayed similar reductions in molecular weight following digestion with peptide: N‐glycosidase F (PNGase F) to remove all N‐glycans (Figure 4D). In contrast to EndoH‐resistant BL EGFRs, a cohort of apically delivered EGFRs were sensitive to EndoH consistent with a Golgi bypass pathway (Figure 4E) [7]. Although the reason for the incomplete EndoH‐sensitive phenotype is unclear, some Golgi‐bypass cargo are thought to be “corrected” post‐delivery, by endocytic trafficking through compartments with low level expression of Golgi‐modifying enzymes [56]. We next tested the effect of treating cells with brefeldin A (BFA) a fungal metabolite specifically inhibiting guanine exchange factors (GEFs) of the Arf family of GTPases [57, 58, 59]. In most cells, BFA blocks COPI coat formation and Golgi secretion by targeting the Arf1 GEF guanylate binding protein 1 (GBF1) [60]. Although MDCK cells possess a BFA‐resistant GBF1 mutation, this drug has additional MDCK cell targets including an ERGIC‐to‐ER retrograde retrieval pathway associated with BFA‐induced Ap secretion of soluble ER cargo such as the chaperone glycoprotein clusterin (Figure 4F, left panels) [61, 62]. The same concentration of BFA effectively blocked EGFR delivery to Ap membranes in TNF‐α‐treated MDCK cells (Figure 4F, right panels) consistent with the drug having a potential impact on EGFR sorting in TGN another well‐characterized MDCK cell BFA target [63, 64, 65]. However, Ap EGFR delivery was not affected by a 20°C temperature block known to inhibit budding of TGN‐derived transport carriers (Figures 4G,H and S4) [66, 67]. Collectively these data supported a working model that EGFRs were trafficked to Ap membranes via an ER‐Ap membrane itinerary independent of passage through ERGIC/Golgi/TGN compartments constituting the classical secretory pathway. Our studies also supported a role for an unidentified BFA‐sensitive Arf family member.

### 2.4 Ligand Binding Triggered a Novel Mode of EGFR Regulation at the Ap Cell Surface

**FIGURE 5:**
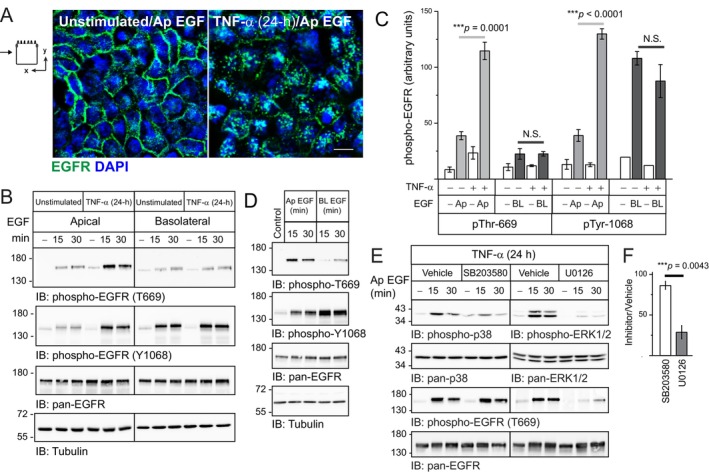
Long‐term TNF‐α exposure stabilized expression of ligand‐stimulated EGFRs initially localized to Ap membranes. (A) Representative cell images from permeabilized cells stained with EGFR antibody (green) and counterstained with DAPI (blue) following a 30‐min stimulation with Ap EGF under unstimulated and TNF‐α‐treated (24 h) conditions. Size bar, 5 μm. (B) Cell lysates were collected from filter‐grown cells following acute domain‐specific EGF stimulation (20 ng/mL) under unstimulated and TNF‐α‐treated conditions. Equal aliquots of total cellular protein (25 μg) were immunoblotted with phospho‐specific (Thr669 and Tyr1068) and pan EGFR antibodies and a tubulin protein loading control antibody. (C) EGFR protein bands in (B) were quantified, phospho‐specific EGFR bands were normalized to their pan‐EGFR antibody controls, and data presented as phospho‐EGFR abundance without and with domain‐specific EGF stimulation in untreated cells and following a 24‐h treatment with TNF‐α (mean ± SEM, *n* = 3). ***p values were 0.0001 and < 0.0001 for comparisons of phospho‐Thr669 and phospho‐1068 levels in cells exposed to Ap EGF respectively (Student's *t* test). (D) Same as (B) except all samples were from TNF‐α‐pretreated cells (24‐h) to facilitate analysis of cells acutely stimulated with Ap versus BL EGF on the same blot. (E) TNF‐α‐treated cells (24‐h) were incubated with DMSO vehicle and pharmacological inhibitors to p38‐MAPK (SB203580; 10 μM) and ERK‐1/2 (U0126; 10 μM) for 1 h prior to acute EGF stimulation (20 ng/mL) at the Ap cell surface carried in the continued presence of MAPK inhibitors. Equal aliquots of total cellular protein (25 μg) were analyzed with phospho‐specific and pan antibodies to p38 and ERK1/2 MAPKs and EGFR. (F) EGFR protein bands in (E) were quantified, phospho‐specific EGFR bands were normalized to their pan‐EGFR reactive control, and data presented as percentage phospho‐Thr669‐labeled EGFR in vehicle versus SB203580‐treated (white bar) and U0126‐treated (grey bar) cells (mean ± SEM, *n* = 3). ***p = 0.0043 (Student's *t* test).

**FIGURE 6:**
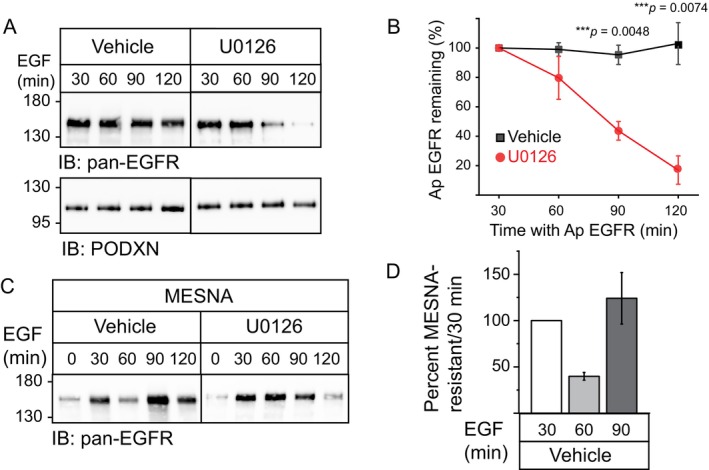
Ligand‐induced Erk1/2 signaling was required for EGFR stabilization on Ap membranes. (A) TNF‐α‐treated cells (24‐h) were incubated with vehicle and U0126 (10 μM) for 1 h, biotinylated at the Ap membrane, and harvested following Ap EGF (20 ng/mL) stimulation in presence of vehicle and U0126. Biotin‐labeled proteins were recovered from cell lysates by SA purification for immunoblot analysis with antibodies to EGFR and the Ap marker PODXN to control for biotin‐labeling efficiency. (B) EGFR protein bands in (A) were quantified and data presented as percent EGFR remaining in cells incubated with vehicle (black lines and symbols) and U0126 (red lines and symbols) as a function of time with the 30‐min post‐EGF stimulation time point set to 100 (mean ± SEM, *n* = 3). ***p = 0.0048 and 0.0074 at 90 and 120‐min time points (Student's *t* test). (C) Same as (A) except cells were labeled with a thiol‐cleavable biotin reagent and cells were harvested after biotin moieties were removed from surface‐labelled proteins by reduction of the disulfide bond with MESNA. (D) EGFR protein bands from vehicle‐treated samples in (C) were quantified and data presented as percent MESNA‐resistant EGFR as a function of time with the 30‐min post‐EGF stimulation time point set to 100 (mean ± SEM, *n* = 3).

To begin determining the biological significance of TNF‐α‐directed trafficking to Ap membranes, the effect of Ap EGF stimulation on EGFR distribution was compared in cells under unstimulated and TNF‐α‐treated conditions. Cell imaging revealed that ligand‐exposed EGFRs were localized in intracellular vesicles consistent with endocytic uptake in cells with up‐regulated Ap EGFR expression associated with long‐term TNF‐α exposure, but not under unstimulated conditions (Figure 5A). We next compared phosphorylation responses to domain‐specific ligand stimulation by immunoblot analysis with phospho‐specific EGFR antibodies recognizing the Tyr1068 autophosphorylation site and phospho‐Thr669 previously linked to EGF‐induced ERK1/2‐MAPK signaling [68]. Both phosphorylation events were enhanced by ligand stimulation of apically localized EGFRs following long‐term TNF‐α exposure (Figure 5B). Interestingly, BL EGF induced phosphorylation at Tyr1068 but had a negligible effect on the Thr669 substrate independent of TNF‐α treatment (Figure 5B). Quantitative analysis confirmed that the relative abundance of Thr669 phosphorylation compared to phospho‐Tyr1068 was indeed significantly higher in TNF‐α‐treated cells stimulated with ligand at Ap versus BL domains (Figures 5C and S6). Equal aliquots of total cellular protein from TNF‐α‐treated cells were resolved on the same SDS‐PAGE gel to facilitate direct comparisons of domain‐specific EGF effects on receptor phosphorylation and corroborate quantitative data (Figure 5D). Ap EGF stimulation studies were repeated following treatment with DMSO vehicle and pharmacological inhibitors targeting p38 and ERK1/2 MAPKs (SB203580 and U0126, respectively) after long‐term TNF‐α treatment. While Ap EGF induced activation of both MAPK family members based on immunoblotting with activation‐specific phospho‐antibodies, its effect on p38‐MAPK was relatively modest compared to ERK1/2‐MAPK (Figure 5E). Additionally, ERK1/2‐MAPK inhibition was associated with a reduction in ligand‐induced phosphorylation at the Thr669 EGFR substrate compared to a negligible effect of the p38‐MAPK inhibitor (Figure 5E). These differences were statistically significant based on comparisons of Thr669 phosphorylation in cells treated with vehicle versus both MAPK family inhibitors (Figures 5F and S7). We next tested the hypothesis that ERK1/2‐MAPK activity modulated post‐endocytic EGFR trafficking based on studies showing ERK1/2‐MAPK‐dependent phosphorylation of Thr669 slowed ligand‐induced down‐regulation in non‐polarized CHO cells [68]. Filter‐grown cells were pre‐incubated with vehicle and U0126 ERK1/2‐MAPK inhibitor for 1 h, biotinylated on the Ap surface, stimulated with Ap EGF, and harvested every 30 min post‐stimulation. SA‐purified proteins were subsequently immunoblotted with antibodies to EGFR and Ap resident PODXN protein to control for biotin labeling. U0126 caused a significant reduction in recovery of surface‐biotinylated EGFRs by 90 min post‐stimulation consistent with ligand‐targeted degradation compared to vehicle (Figures 6A,B and S8). These studies were repeated using a membrane impermeant labeling reagent with a thiol‐cleavable biotin moiety. EGF‐stimulated cells were subsequently harvested after surface‐bound biotin was removed using the cell‐impermeable reducing agent 2‐mercaptoethane sulfonate sodium (MESNA). These experiments revealed MESNA‐resistant EGFRs accumulated intracellularly within the first 30 min of Ap EGF stimulation under both treatment conditions. Similar to total Ap EGFR levels, the intracellular EGFR pool was diminished over time following pharmacological ERK1/2 inhibition (Figure 6C). However, EGFRs re‐acquired MESNA sensitivity by 60 min post‐stimulation followed by another apparent round of internalization consistent with EGFR recycling in vehicle‐treated cells (Figures 6C,D and S8). Altogether, these results supported a working model that ligand‐stimulated ERK1/2‐MAPK activity slowed EGFR degradation and promoted recycling after receptors were internalized from the Ap membrane in TNF‐α stimulated cells.

### 2.5 Ligand Binding Induced PI3K/Akt Signaling From Ap Membrane Compartments

**FIGURE 7:**
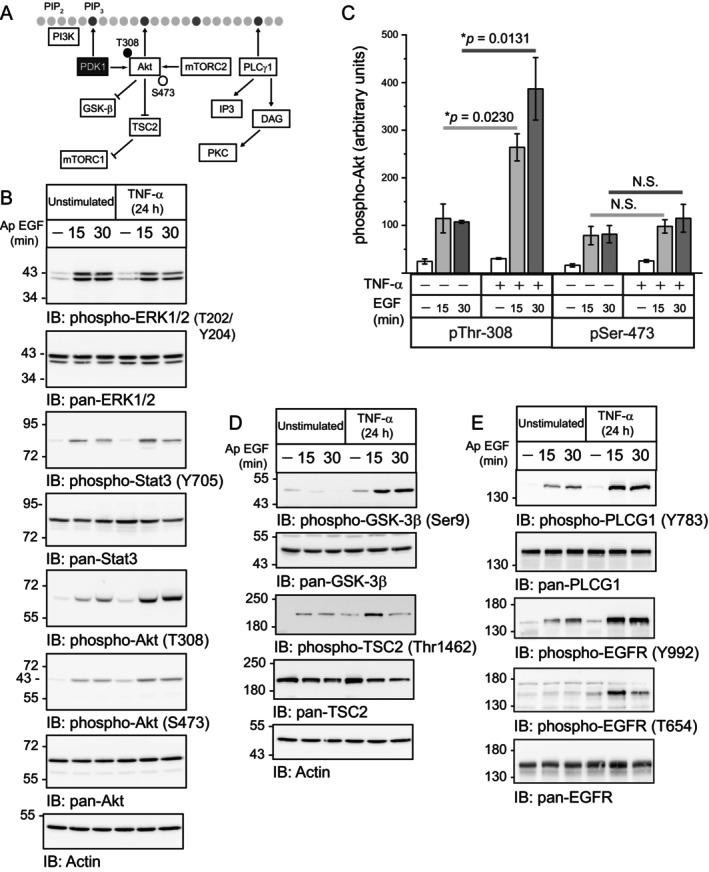
TNF‐α‐mediated EGFR localization to Ap membranes selectively enhanced ligand‐induced PI3K/Akt signaling. (A) Schematic summarizing signaling responses associated with Akt activation and regulation by PLCG1/PKC‐α downstream of PI3K‐generated PIP3 docking sites. (B) Filter‐grown cells were acutely stimulated with EGF (20 ng/mL) from the Ap membrane under unstimulated and TNF‐α‐treated conditions. Equal total cell protein aliquots (25 μg) were immunoblotted with phospho‐specific and pan antibodies to signaling molecules listed in the figure. (C) Akt protein bands in (B) were quantified, phospho‐specific Akt proteins were normalized to input controls, and data presented as phospho‐Akt abundance without and with Ap domain‐specific EGF stimulation following a 24‐h treatment with TNF‐α (mean ± SEM, *n* = 3). *p‐values were 0.0230 and 0.0131 for comparisons of phospho‐Thr308 levels at 15‐ and 30‐min post Ap EGF stimulation (Student's *t* test). (D and E) Same as (A) except samples were immunoblotted with phospho‐specific and pan antibodies to Akt substrates GSK‐3β and TSC2 and an Actin protein loading control (D); and phospho‐specific antibodies to PLCG1, the EGFR autophosphorylation PLCG1 docking site Tyr992, the EGFR PKC‐dependent substrate Thr654, and pan PLCG1 and EGFR antibodies (E).

**FIGURE 8:**
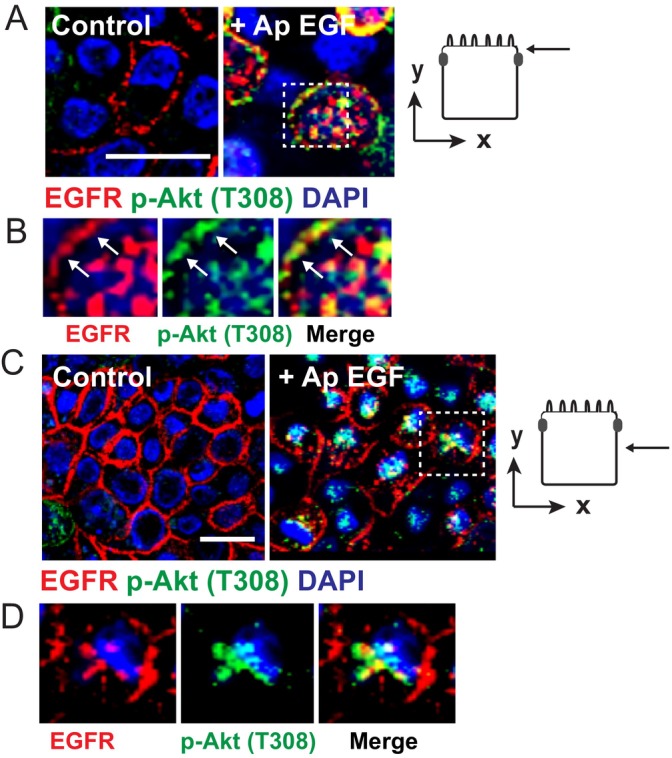
Phospho‐Akt (Ser308) was localized to cellular membrane compartments following stimulation of TNF‐α‐treated cells with Ap EGF. (A and B) Representative images from the Ap side of tight junction adhesion proteins of filter‐grown cells (see icon to right) treated with TNF‐α for 24‐h under unstimulated and Ap EGF‐stimulated conditions. Fixed and permeabilized cells were co‐stained for EGFR (red) & phospho‐Akt (T308) (green) (A). Boxed area shows region of interest in the EGF‐stimulated image that was magnified 2× to visualize individual and merged channels with arrows denoting Ap membrane margins (B). (C and D) Same as (A and B) except images were from midpoint of lateral surface of filter‐grown cells (see icon to right).

We continued analyzing the potential biological significance of TNF‐α‐directed trafficking to Ap membranes by focusing on signaling pathways with distinctive domain‐specific responses to acute EGF stimulation described in Figure 1D, particularly those involving PI3K signaling (Figure 7A). TNF‐α‐mediated enhancement of Ap EGFR expression did not appear to alter acute ERK1/2‐MAPK and Stat3 responses to stimulation with Ap ligand (Figure 7B). However, there were notable differences in PI3K‐Akt signaling following long‐term TNF‐α exposure compared to unstimulated conditions (Figure 7B). Akt is phosphorylated at two sites downstream of EGFR‐PI3K signaling, Thr308 and Ser473, modified by PDK1 (Phosphoinositide‐Dependent Kinase‐1) and mTORC2 (mTOR Complex 2) respectively (Figure 7A) [69]. While Ap ligand induced increased levels of phospho‐Thr308, its effect on phospho‐Ser473 was negligible (Figure 7B). These differing responses displayed a time‐dependent increase in statistical significance when levels of pThr308‐Akt and pSer473‐Akt proteins were compared following stimulation of TNF‐α‐treated cells with Ap EGF compared to untreated conditions (Figures 7C and S9). Our results prompted us to examine a subset of Akt substrates, GSK‐3β (glycogen synthase kinase‐3 beta) and TSC2 (tuberous sclerosis complex) also known as tuberin (Figure 7A) [70]. GSK‐3β and TSC2 both exhibited increased phosphorylation at their respective Akt substrates in cells with increased Ap EGFR expression (Figure 7D). The TSC2 modification was comparatively short‐lived, consistent with its documented phosphorylation‐dependent degradation downstream of PI3K‐Akt signaling (Figure 7D) [71]. We also examined the PLCG1 pathway since PLCG1 is known to translocate to the plasma membrane via a PIP3‐binding pleckstrin homology domain located at its N‐terminus by a mechanism previously linked to PI3K signaling (Figure 7A) [72]. PLCG1 and the EGFR autophosphorylation docking site Tyr992 [73] both displayed increased phosphorylation levels following ligand stimulation of cells with enhanced Ap EGFR expression (Figure 7E). We also observed a spike in EGFR phosphorylation at its protein kinase C (PKC) substrate Thr654, a potential target for the diacylglycerol (DAG)/PKC branch of the PLCG1 signaling pathway (Figure 7E) [74, 75, 76]. Finally, cell imaging studies revealed that phospho‐Akt (Thr308) was detectable in membrane compartments in TNF‐α‐treated cells stimulated with Ap EGF. These compartments were observed in Z‐stack images at limiting membranes located on the Ap side of the tight junctional adhesion barrier [Figure 8A,B (see arrows)] and the perinuclear region (Figure 8C,D). The phospho‐Akt (Thr308)‐positive compartments did not significantly overlap with EGFR signals, consistent with reports that Akt, which does not physically associate with EGFR, may be activated by receptors that are internalized and progressing through endosomal compartments [77, 78]. We concluded that long‐term TNF‐α exposure selectively induced PI3K signaling by subverting EGFR trafficking to Ap membranes, potentially affecting multiple downstream targets in fully polarized MDCK cells.

## 3 Discussion

Studies over the past few decades have highlighted the complex interrelatedness between the pro‐inflammatory cytokine TNF‐α and EGFR. Ligand‐stimulated TNFR1 and EGFR pathways converge on common downstream signaling targets, resulting in both synergistic and antagonistic effects influencing multiple cellular processes [79, 80]. TNF‐α is also one of a growing number of p38‐MAPK‐inducing cellular stresses capable of triggering non‐canonical EGFR trafficking and signaling independent of ligand binding [45]. In this manuscript, we have described an unexpected and new role for TNF‐α in up‐regulating EGFR function from Ap membrane compartments in polarized epithelial cells. Interestingly, not all signaling pathways were amplified by activating receptors from Ap membranes, underscoring an underappreciated role for cell polarity in fine‐tuning EGFR responses. Our results demonstrated that long‐term TNF‐α exposure changed the direction and speed of polarized EGFR sorting, allowing for rapid engagement of ligand‐dependent EGFR/PI3K signaling from the Ap membrane. Our studies provide new opportunities for understanding how innate immunity dynamically co‐opts molecular mechanisms controlling polarized EGFR trafficking and possible roles for PI3K signaling in immune sensing by barrier epithelial cells.

PI3K phosphorylates phosphatidylinositol‐4,5‐bisphosphate (PIP2) to synthesize phosphatidylinositol‐3,4,5‐trisphosphate (PIP3) creating docking sites for recruiting numerous signaling molecules to cell membranes, including the serine/threonine‐specific protein kinase Akt [69]. Not only is it abundantly expressed on Ap membranes, but PIP2 is the principal organizer of Ap membrane biogenesis in MDCK cells [81, 82]. Until recently, it was generally thought that Akt activation required simultaneous phosphorylation events at Thr308 in its kinase activation loop and Ser473 in a C‐terminal hydrophobic motif by the PI3K‐regulated enzyme PDK1 and mTORC2, respectively [83]. However, mounting evidence suggests that phospho‐Thr308 may be the better predictor of Akt activity under normal physiological conditions and oncogenic functions in several human cancers [84, 85, 86]. Interestingly, Ap EGF enhanced Akt phosphorylation at Thr308 but not Ser473 in TNF‐α‐treated cells, suggesting PDK1 may be preferentially recruited onto Ap plasma membranes. We also examined a limited subset of Akt substrates, demonstrating that phospho‐Thr308 was associated with enhanced Akt activity in TNF‐α‐treated cells stimulated with Ap EGF.

We are currently investigating mechanisms preferentially linking EGFRs to downstream PI3K signaling events from Ap membranes. EGFRs lack an autophosphorylation docking site for PI3K but can activate this enzyme indirectly through interactions with multiple adaptor proteins including Akt kinase‐interacting protein 1 (Aki1), Grb2‐associated binder 1 (Gab1), Src homology 2 domain‐containing transforming protein C1 (Shc1), and IQ motif containing GTPase activating protein 1 (IQGAP1) following ligand stimulation [87, 88, 89, 90, 91]. EGFR/IQGAP1 complexes are known to be mainly distributed on BL membranes and associated with mitotic spindle misalignment when erroneously recruited to Ap membranes via interactions with missorted EGFR [92]. In contrast, relatively little is currently known about how cell polarity contributes to the regulation of other adaptors linking EGFR to PI3K/Akt. In addition to potential roles for various adaptor proteins, Ap EGFRs could activate PI3K through its kinase‐dead heterodimer partner ErbB3 which directly binds PI3K following EGF stimulation [93, 94]. ErbB3 is expressed in MDCK cells where it is presumably localized on BL membranes as seen in other polarized epithelia [95, 96]. However, TNF‐α exposure could foster EGFR‐ErbB3 heterodimer formation in response to Ap EGF ligand by having similar effects on the polarized trafficking of both ErbB family members.

It's increasingly recognized that membrane compartmentalization is a key factor controlling substrate specificity of PI3K/Akt signaling [78]. Related questions have so far been studied primarily in the context of cancer cells but remain poorly understood in polarized epithelial cells. Our studies have suggested that Akt signals were propagated from intracellular membranes after endocytosis of activated EGFRs in line with multiple studies indicating that EGFR/PI3K signaling is not confined to the plasma membrane [97, 98]. We have also shown that apically internalized EGFRs appeared to be recycled back to the Ap membrane although the exact itinerary is not currently known. In general, endocytosed Ap cargo is initially sorted to Ap early endosomes (AEE) and from there can be rapidly recycled, delivered to lysosomes for degradation, or sorted to the common recycling endosome (CRE) where apically targeted proteins are subsequently retrieved to the Ap recycling endosome (ARE) on route back to the Ap membrane [99]. The complexity of the endocytic machinery is an underappreciated factor contributing to the spatial organization of PI3K/Akt signaling in polarized epithelial cells.

**FIGURE 9:**
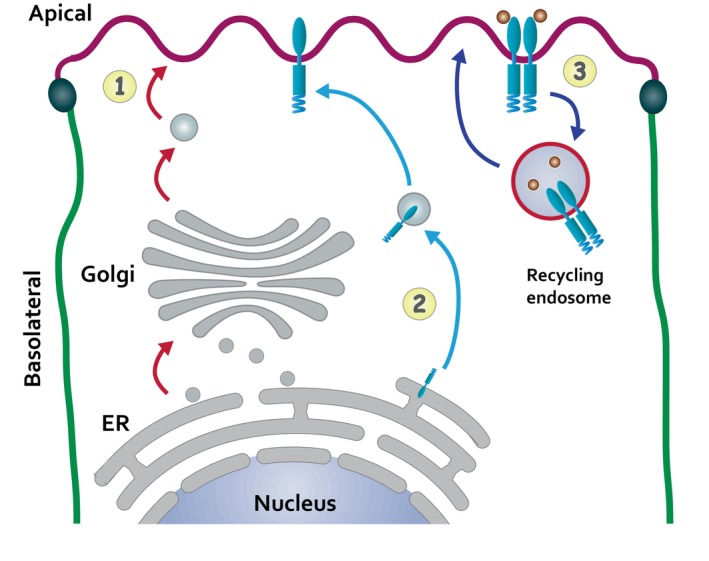
Summary model. Schematic showing classical Ap secretory pathway (1, red arrows). Our studies have identified putative pathways regulating unconventional EGFR secretion (2, turquoise arrows), and recycling of internalized EGFR back to the Ap membrane following ligand stimulation (3, dark blue arrows). See text for further discussion.

Our previous studies have shown that EGFRs are targeted to BL membranes via a conventional secretory pathway under regulation of the epithelial‐specific AP1B clathrin adaptor [26, 28]. Long‐term TNF‐α treatment did not have an apparent effect on this sorting route. In contrast, TNF‐α promoted a transport pathway where EGFR reaches the Ap membrane extremely rapidly without passage through the Golgi complex, presumably via a mechanism involving peripheral components of the ER (Figure 9). Molecular mechanisms linking TNF‐α signaling to this novel EGFR trafficking pathway are an important topic for future investigation. Several COPII components regulating EGFR transport in the canonical pathway are known to be up‐regulated by the endosomal transcriptional regulator RNF11 to re‐populate plasma membrane receptor pools following ligand‐induced EGFR degradation [100]. A TNF‐α‐mediated transcriptional program could play a similar role regulating the direction and speed of EGFR transport along a novel Golgi bypass pathway. We are also considering a potential role for an ER‐associated degradation (ERAD)‐like mechanism. Originally identified because of key roles in the unfolded protein response (UPR), ERAD sensing pathways are increasingly recognized as physiological targets for multiple signaling molecules [101]. Additionally, a variety of studies have shown that ER stress responses are induced by inflammatory cytokines and have important roles regulating immune responses in both physiological and pathological settings [102]. We have also demonstrated that luminal TNF‐α was capable of transmitting signals to BL EGFRs in filter‐grown MDCK monolayers. This sets up a possible scenario in which EGFR exerts feedback control over its trafficking itineraries in response to innate immune sensing across an epithelial barrier. TNF‐α and EGF are both known to induce transient and moderate activation of the inositol‐requiring enzyme type 1α)/ X‐box binding protein 1 (IRE1α/XBP1) branch of the ER stress response independent of a conventional UPR by mechanisms that warrant further investigation: TNF‐α by a Reactive Oxygen Species (ROS)‐dependent mechanism that is cytoprotective [103]; and EGF via EGFR/PLCG1 (phospholipase Cg1)/IP3 (inositol triphosphate) signaling, which opens IP3R Ca^2+^‐release channels in the ER [104].

EGFR is phosphorylated at multiple serine and threonine residues, contributing to the regulation of various receptor functions, including dimerization, catalytic activity, and protein stability [105]. Thr669, located in the receptor's cytoplasmic juxta‐membrane region was initially recognized as a prominent ERK1/2‐MAPK phosphorylation target following ligand stimulation [74, 106]. Since then, it has emerged as a critical check point controlling a molecular switch between constitutive and ligand‐regulated EGFR signaling, by inhibiting unstimulated signaling in EGFR overexpressing cancer cells and enhancing EGFR stability and signaling following ligand stimulation in non‐polarized cell models, respectively [68, 107]. We found that Thr669 was preferentially phosphorylated on apically localized EGFRs by a mechanism requiring ligand‐stimulated ERK1/2‐MAPK activity following long‐term TNF‐α exposure. Additionally, EGF‐induced downregulation of Ap EGFR abundance from intracellular pools was much more rapid in cells treated with an ERK1/2‐MAPK pharmacological inhibitor versus a vehicle control. These findings supported a working model that ligand‐induced ERK1/2 activity exerts positive feedback control over EGFR/PI3K signaling from Ap membranes (Figure 9). Low levels of Ap EGFR found in untreated cells were associated with significant ERK1/2‐MAPK activation upon ligand stimulation. However, the finding that phospho‐Thr‐669 was preferentially seen following long‐term TNF‐α‐exposure suggested the cytokine regulated how downstream EGFR signaling pathways are organized as well as EGFR abundance on Ap membranes. Possible mechanisms currently being investigated include partitioning into specialized signaling compartments and/or recruitment of unique molecular scaffolds coordinating ERK1/2‐MAPK and PI3K activation. Interestingly, we've shown previously that BL EGFR localization is controlled in part by a proline‐based signal including Thr669, suggesting phospho‐Thr669 could contribute to Ap EGFR sorting by disrupting the receptor's interaction with BL sorting machinery [29].

TNF‐α is a major regulator of acute phase and systemic inflammatory responses and a contributing factor in the pathogenesis of multiple chronic inflammatory diseases [108]. It is also an abundant inflammatory cytokine in the tumor microenvironment with mechanistic links to cancer growth and metastasis [109]. Our studies have revealed a potential new mechanism by which TNF‐α from activated immune cells regulates epithelial cell behavior by promoting EGFR/PI3K/Akt signaling from Ap membranes. Prior studies established that Akt is a downstream target for TNF‐α/TNFRI signaling with an essential role in NFκB activation in several cell models, including epithelial‐derived breast cancer cells [110, 111]. It will now be interesting to see how TNF‐α employs the same pathway to perform distinct tasks related to inflammatory signaling in barrier epithelial cells. MDCK cells are well‐suited for establishing fundamental cell biological processes, particularly diverse intracellular pathways regulating polarized secretion. However, it is now important to extend these studies to physiological epithelial cell models with native EGFR expression involved in innate immune sensing to fully appreciate the role of the EGFR/PI3K/Akt axis in inflammatory signaling in the molecular context of the Ap membrane.

## 4 Materials and Methods

### 4.1 Cells and Primary Antibodies and Reagents

Strain II MDCK (MDCK‐II) [CLS Cat# 602280/p823_MDCK_(NBL‐2), RRID:CVCL_0422] is a spontaneously immortalized non‐transformed canine kidney epithelial cell line with a hyper‐diploid karyotype lacking consistent identifiable marker chromosomes obtained from the American Type Culture Collection (ATCC catalog number CCL34) [112]. A stable MDCK‐II cell line with CMV promoter‐driven human wild‐type EGFR (WT‐EGFR) transgene was described previously [28, 29, 43]. MDCK cells were maintained with Eagle's minimal essential medium (EMEM) (Cytiva catalog number10‐009‐CV) supplemented with 10% HyClone characterized fetal bovine serum (FBS) (Cytiva catalog number SH30071.03). Cells were screened for mycoplasma contaminants using a PCR‐based Universal Mycoplasma Detection Kit from ATCC (catalog number 30–1012 K) and cultured no more than 15 passages after initial thawing. Actively growing cells were seeded on polycarbonate Transwell filter inserts (0.4 μm pore size) at 2.0 × 10^5^ cells/cm^2^ and re‐fed every other day for the duration of experiments. Cell counts were determined using a Scepter 3.0 handheld automated cell counter (Millipore). See Table 1 for a comprehensive list of primary antibodies used in this study. Anisomycin (catalog number 222), BFA (catalog number 9972), recombinant human EGF (catalog number 7258), SB203580 (catalog number 5633), and U0126 (catalog number 9903) were from Cell Signaling Technology. Recombinant canine IL‐8 (catalog number 1608‐CL) and recombinant canine TNF‐α (catalog number 1507‐CT) were from R&D Systems.

**TABLE 1:** Antibodies used in the study.

Target	Source	Vendor	Catalog #	RRID
Actin	Mouse	CST^a^	3700	AB_2242334
Akt	Rabbit	CST	9272	AB_329827
Akt (pThr308)	Rabbit	CST	4056	AB_331163
Akt (pSer473)	Rabbit	CST	4060	AB_2315049
CXCR1	Rabbit	Proteintech	55 450‐1‐AP	AB_11182706
Clusterin (biotin)	Rabbit	US Biologicals	546 019	
E‐cadherin	Mouse	BD Biosciences	610 181	AB_397580
EGFR	Rabbit	CST	4267	AB_2895042
EGFR	Mouse	Ascites^b^		
EGFR (pThr654)	Mouse	Abcam	ab78283	AB_1566154
EGFR (pThr669)	Rabbit	CST	8808	AB_11179086
EGFR (pTyr992)	Rabbit	CST	2235	AB_331708
EGFR (pSer1046/47)	Rabbit	CST	2238	AB_331129
EGFR (pTyr1068)	Rabbit	CST	3777	AB_2096270
ERK 1/2	Rabbit	CST	9102	AB_330744
ERK 1/2 (pThr202/pTyr204)	Rabbit	CST	4370	AB_2315112
GSK‐3b	Rabbit	CST	12 456	AB_2636978
GSK‐3b (pSer9)	Rabbit	CST	5558	AB_10013750
JNK	Rabbit	CST	9252	AB_2250373
JNK (pTyr183/Tyr185)	Rabbit	CST	4668	AB_823588
p38‐MAPK	Rabbit	CST	8690	AB_10999090
p38‐MAPK (pThr180/Tyr182)	Rabbit	CST	4511	AB_2139682
PLCG1	Rabbit	Aviva		ARP33086
PLCG1 (pTyr783)	Rabbit	CST	14 008	AB_2728690
PODXN	Mouse	DSHB^c^	3F2/D8	AB_2618385
Stat3	Rabbit	CST	4904	AB_331269
Stat3 (pTyr705)	Rabbit	CST	9145	AB_2491009
TAMRA	Mouse	Thermo‐Fisher	MA1‐041	AB_2536728
TNFR1	Rabbit	CST	3736	AB_2241018
TSC2	Rabbit	CST	4308	AB_10547134
TSC2 (pThr1462)	Rabbit	CST	3617	AB_490956
Tubulin	Rabbit	CST	2125	AB_2619646

^a^ CST, Cell Signaling Technology.

^b^ Induced by EGFR1 hybridoma cell line.

^c^ Developmental Studies Hybridoma Bank (DSHB) developed under the auspices of NICHD and maintained by The University of Iowa Department of Biological Sciences (Iowa City, IA).

### 4.2 Cell Harvesting, Immunoprecipitation, and Immunoblotting

Cells were rinsed 3 times with Dulbecco's‐modified phosphate‐buffered saline (DPBS) supplemented with 5 mM EDTA, 5 mM EGTA, 10 mM NaF, and 10 mM Na_4_P_2_O_7_. Cells were harvested with lysis buffer supplemented with a protease/phosphatase inhibitor cocktail (Cell Signaling Technology catalog numbers 9803 and 9803 respectively) for 30 min on ice. Immunoprecipitations were carried out using antibodies adsorbed to protein G‐agarose beads (Pierce catalog number 20398). Antibody‐specific immune complexes and equal protein aliquots of input whole cell lysate determined by linear regression analysis of a BSA standard curve in a Bradford assay were resolved by SDS‐PAGE and transferred to nitrocellulose membranes using standard methods. Nitrocellulose membranes were blocked with Tris‐buffered saline supplemented with 1% Tween (TBST) and 5% non‐fat dry milk (NFDM). Blots were incubated with primary antibodies diluted in TBST‐NFDM overnight at 4°C, followed by a 1‐h incubation with HRP‐conjugated secondary antibodies (Cell Signaling Technology) diluted in TBST‐NFDM at room temperature for detection by enhanced chemiluminescence using the ECL Prime Western Blotting System from Cytiva (catalog number RPN2232). Chemiluminescent signals were captured using an ImageQuant LAS 4000 charged couple display (CCD) camera system (GE Health Sciences). Figures show representative blots from at least three independent experiments. Sub‐saturated protein bands of interest and background measurements below each protein band, which were deducted from protein band values, were quantified with the ImageJ processing program from the National Institutes of Health.

### 4.3 Domain‐Specific Biotin Labeling of Cell Surface Proteins

Filter‐grown cells were rinsed three times with DPBS supplemented with 1 mM CaCl_2_ and 1 mM MgCl_2_ (DPBS‐CM), followed by a 30‐min domain‐specific incubation with the amine‐reactive and membrane‐impermeable reagent N‐hydroxysulfosuccinimidobiotin (1 mg/mL DPBS‐CM) (Thermo‐Fisher catalog number 21217) at 4°C. The biotinylation reaction was quenched by rinsing cells three times with 50 mM NH_4_Cl prior to cell harvesting. For “MESNA‐resistance” assays [113], cells were rinsed three times with DPBS‐CM followed by a 30‐min incubation with the thiol‐cleavable, amine‐reactive, and membrane‐impermeable biotinylation reagent sulfo‐NHS‐SS‐biotin (1 mg/mL DPBS‐CM; Thermo‐Fisher catalog number 21331) at 4°C. The reaction was quenched by three rinses with HEPES‐buffered EMEM supplemented with 10% FBS. Cells were harvested without further treatment, and after surface‐bound biotin was removed using the cell‐impermeable reducing agent 2‐mercaptoethane sulfonate sodium (MESNA; 50 mM; Sigma‐Aldrich catalog number M1511) in a solution of 50 mM Tris, pH 8.6, supplemented with 0.1 M NaCl, 1 mM MgCl_2_, and 0.1 mM CaCl_2_ (2 × 20 min incubations on ice). Excess MESNA was quenched by a 15‐min incubation with iodoacetamide (5 mg/mL in DPBS‐CM) on ice, and cells were lysed as previously described. Biotinylated proteins were subsequently affinity‐purified on SA‐agarose beads (Invitrogen catalog number S951) for SDS‐PAGE and immunoblot analysis.

### 4.4 Pulse‐Chase Metabolic Labeling Using Copper‐Free Click Chemistry

Membrane domain‐specific delivery of newly synthesized EGFR was evaluated by modifying a recently published pulse‐chase labeling method employing copper‐free click chemistry [51] (see Figure 4A). Filter‐grown cells were rinsed twice and pre‐incubated for 30 min with Met‐free RPMI media (Gibco catalog number A1451701) supplemented with 5% dialyzed FBS (dFBS). Met‐starved cells were labeled for 30 min from the BL surface, by incubating 24‐mm filters in 500 μL of Met‐free RIPA/5% dFBS containing 50 μΜ of the azide‐modified met analog AHA (L‐azidohomoalanine) (Invitrogen catalog number C10102) cell‐side up in parafilm‐lined bacterial dishes. Filters were returned to 6‐well dishes and rinsed 2 times and then incubated with complete media supplemented with 50 μΜ unlabeled met for various times. AHA‐labeled cells were rinsed three times with ice‐cold MEM supplemented with 25 mM HEPES, pH 7.4, and 1% BSA (M/H/B), incubated with EGFR1 ascites (10 μL/mL) recognizing a surface‐exposed EGFR epitope [114] added to one side of the filter for 1 h on ice, and then lysed for recovery of EGFR immunoprecipitants on protein G beads. Immunoprecipitants were washed twice with lysis buffer and twice with PBS to remove residual detergent. Washed beads were incubated with a 5 μΜ solution of the azide‐reactive TAMRA‐DBCO probe (Vector Laboratories catalog number CTT‐A131) diluted in PBS supplemented with protease/phosphatase inhibitors for 1 h at room temperature with gentle rotation. TAMRA‐reacted immunoprecipitants were resolved by SDS‐PAGE for immunoblot analysis with TAMRA monoclonal antibody 5G5 (Invitrogen catalog number PIMA1041) and a secondary mouse IgG1‐specific HRP reagent (Cell Signaling Technology catalog number 96714). AHA and TAMRA‐DBCO were prepared as 1000x stocks in DMSO and stored as aliquots at −20°C.

### 4.5 Endoglycosidase Digestions

Newly delivered cell surface EGFR was digested with recombinant peptide: N‐glycosidase F (PNGase F) (New England Biolabs catalog number P0704) and Endoglycosidase H (EndoH) (Sigma‐Aldrich catalog number 324717) according to the manufacturer's instructions. Briefly, AHA‐labeled and TAMRA‐reacted EGFR1 immunoprecipitants were resuspended in 36 μL ddH_2_0 and 4 μL of a 10× denaturing solution and heated for 5 min in a 95°C water bath. Samples were allowed to cool to room temperature for 5 min, and protein G beads were pelleted by maximum speed centrifugation for 10 min in a table‐top microfuge. Eighteen μL of supernatant were transferred to a fresh microfuge tube with 2 μL of 10× reaction buffer. Samples were incubated without (control) and with (digested) approximately 500 units endoglycosidase for 18 h in a gently shaking 37°C water bath. Control and digested samples were resolved by SDS‐PAGE for immunoblot analysis with a rabbit EGFR antibody recognizing a cytosolic determinant.

### 4.6 Cell Imaging

Filter‐grown cells were rinsed 3 times with PBS supplemented with 1 mM CaCl_2_ and 1 mM MgCl_2_ (PBS‐CM), permeabilized with 0.5% saponin in a solution of 80 mM PIPES, pH 6.8, supplemented with 5 mM EGTA and 1 mM MgCl_2_ for 5 min, and fixed with 3% paraformaldehyde–PBS for 15 min [115]. Transwell filter supports were excised from plastic inserts, blocked with a solution containing 1% normal serum from the host animal used to generate secondary antibodies for 30 min at room temperature, and sequentially stained with primary and highly cross‐absorbed secondary antibodies diluted in a solution containing 0.5% saponin and 3% ultra‐pure BSA at 4°C overnight and 1 h at room temperature, respectively. Nuclei were visualized by the addition of 4',6‐diamidino‐2‐phenylindole dihydrochloride (DAPI) or Hoechst 33342 (1 mg/mL) during the secondary antibody staining step. All staining reagents were purchased from Invitrogen Molecular Probes. Filters were mounted cell‐side up on a glass slide and covered with a glass coverslip (1½ thickness) using ProLong Gold Antifade Mountant (Invitrogen catalog number P36980). Cell images were acquired as *Z*‐stacks in the range of 0.4–0.6 ∝μ thickness at room temperature on a fixed platform using a digital EVOS M5000 imaging system (Invitrogen) with 3‐color fluorescence (357/447 nm, 470/525 nm, and 585/624 nm) and transmitted‐light capabilities, and multiple objectives including an Olympus Plan‐Apochromat 63×/1.4 oil DIC objective. Images were analyzed using Celleste Image Analysis Software with modules for 2D deconvolution and 3D reconstructions of *Z*‐stack series (Invitrogen).

### 4.7 TEER And Paracellular Permeability Measurements

Trans‐epithelial electrical resistance (TEER) was measured at 37°C using an EVOM epithelial voltohmmeter with an STX‐2 electrode (World Precision Instruments). TEER values were calculated by subtracting the background TEER of blank filters, normalized to the area of the monolayer, and data expressed as ohms/cm^2^. Paracellular permeability was assessed by measuring the flux of fluorescein‐dextran conjugates (molecular weights of 3 and 40 kDa; Invitrogen catalog numbers D3305 and D1844 respectively). Dextran conjugates were dissolved in 10 mM HEPES, pH 7.4, supplemented with 1 mM sodium pyruvate, 10 mM glucose, 3 mM CaCl_2_, and 145 mM NaCl (reconstitution buffer) at a concentration of 10 mg/mL. Cells were rinsed 3 times and allowed to equilibrate in reconstitution buffer for 10 min. Apical cell culture media were replaced with dextran conjugate solutions, and the amounts of dextran conjugates in the BL compartment were measured using a BioTek Synergy HTX Multimode Reader (Agilent) in the fluorescence modality [Ex (nm) 494 nm, Em (nm) 521] after a 30‐min incubation at 37°C. A standard curve was used to convert relative fluorescent units to the concentration of dextran in the BL chamber (nmol/cm^2^). All measurements were made in triplicate in at least three independent experiments.

### 4.8 Statistical Methods

All quantitative data were derived from three to four biological replicates and presented as the mean ± SEM. Comparisons between two groups were conducted using a two‐tailed student's *t* test, and statistically significant *p* values were represented by one (< 0.05), two (< 0.01), or three (< 0.001) asterisks. All data were analyzed using GraphPad Prism version 6.0.

## Conflicts of Interest

The authors declare no conflicts of interest.

## Supporting information


**Supplemental Figure S1.** Raw data related to quantitative western blot analysis in Figure 1B,G.

**Supplemental Figure S2.** Raw data related to quantitative western blot analysis in Figure 2C,D.

**Supplemental Figure S3.** Raw Z‐stack images related to Figure 2F.

**Supplemental Figure S4.** Raw data related to quantitative western blot analysis in Figure 4C,H.

**Supplemental Figure S5.** Pulse‐chase analysis of domain‐specific EGFR delivery in untreated cells.

**Supplemental Figure S6.** Raw data related to quantitative western blot analysis in Figure 5C.

**Supplemental Figure S7.** Raw data related to quantitative western blot analysis in Figure 5F.

**Supplemental Figure S8.** Raw data related to quantitative western blot analysis in Figure 6B,C.

**Supplemental Figure S9.** Raw data related to quantitative western blot analysis in Figure 7C.

**Data S1.** Supporting Data.

**Supplementary Table 1.** Comprehensive list of primary antibodies used in this study.

## Data Availability

The data underlying this study are available in the published article and its online Supporting Information.
