# Supplementary figures and images for: TNF‐α‐Driven Changes in Polarized EGF Receptor Trafficking Facilitate Phosphatidylinositol 3‐Kinase/Protein Kinase B Signaling From the Apical Surface of MDCK Epithelial Cells
Source: Traffic. 2025 May 5;26(4-6):e70005. doi: 10.1111/tra.70005 (PMC12052438; doi:10.1111/tra.70005)

Figure 1B, 1G raw data

Figure 1B

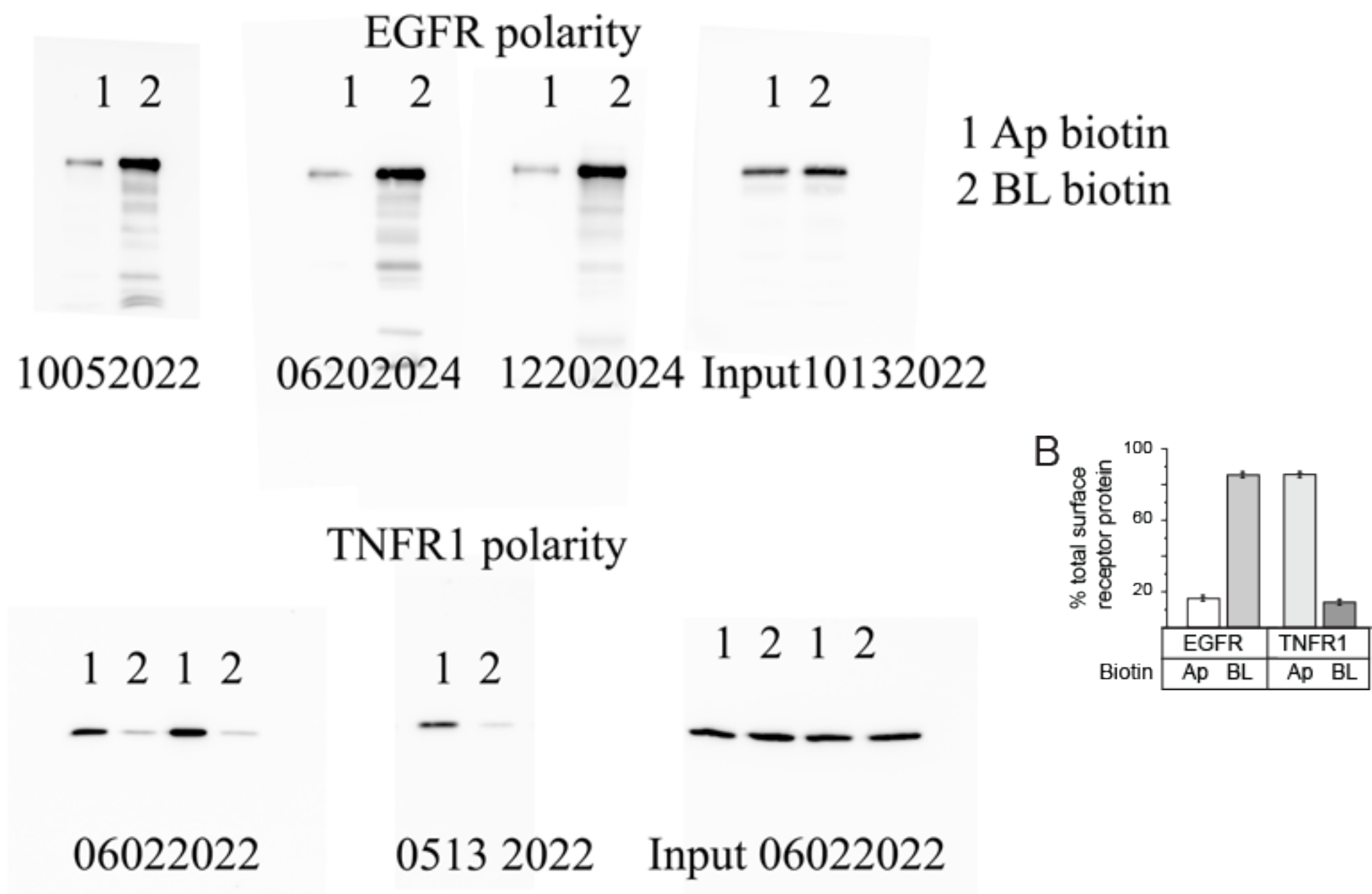

Figure 1G

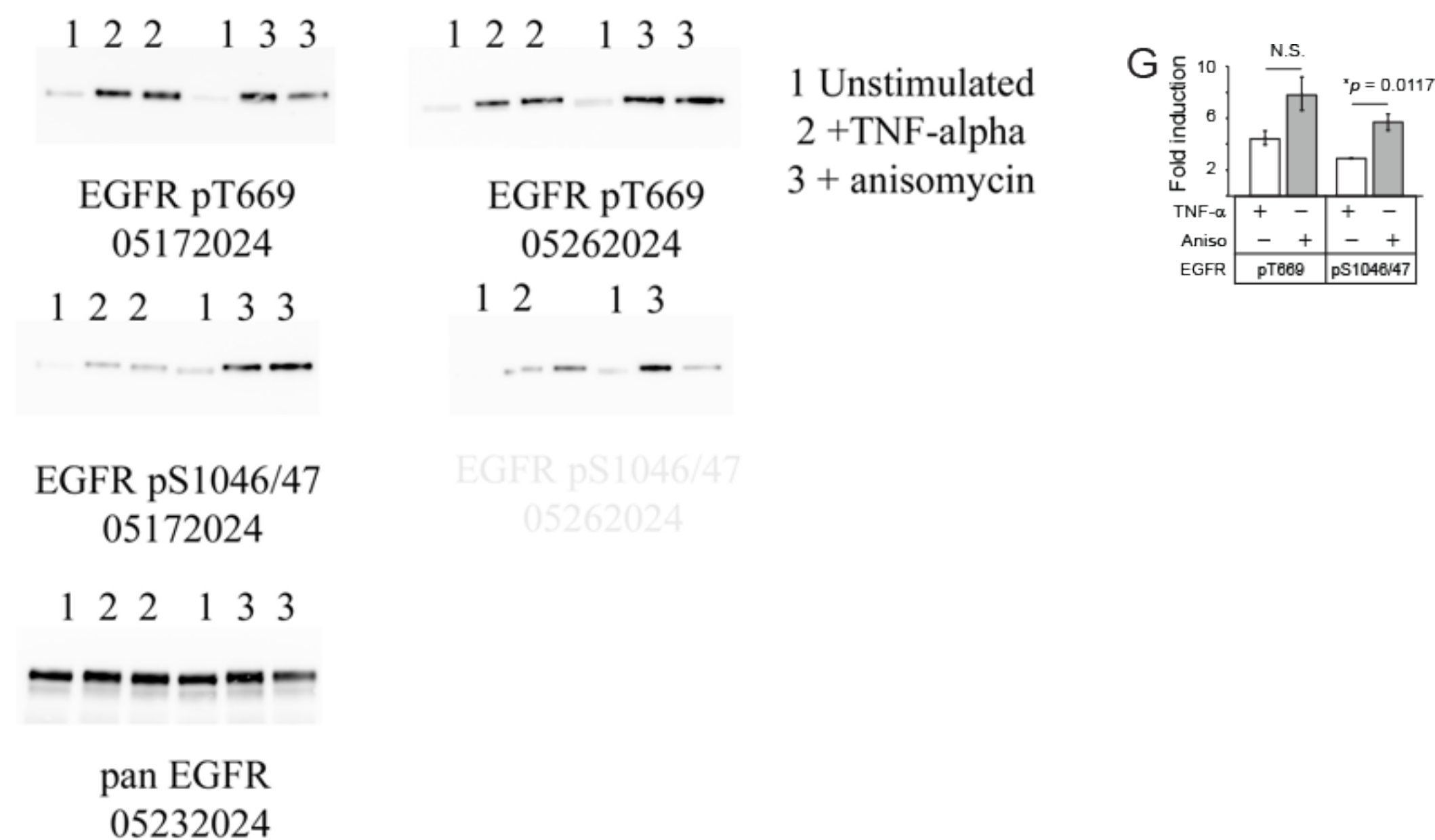

Supplement: Supplementary file 1 — Supplemental Figure S1. Raw data related to quantitative western blot analysis in Figure 1B,G. [file TRA-26-e70005-s008.pdf]

Figure 2C, 2D

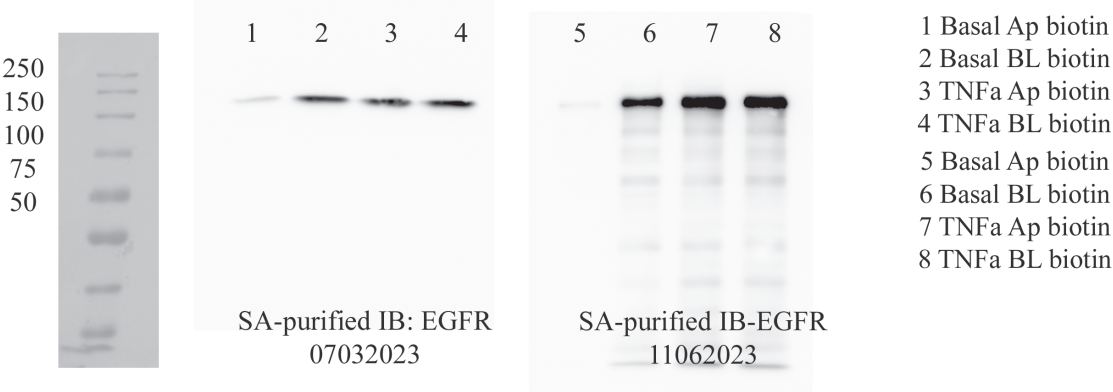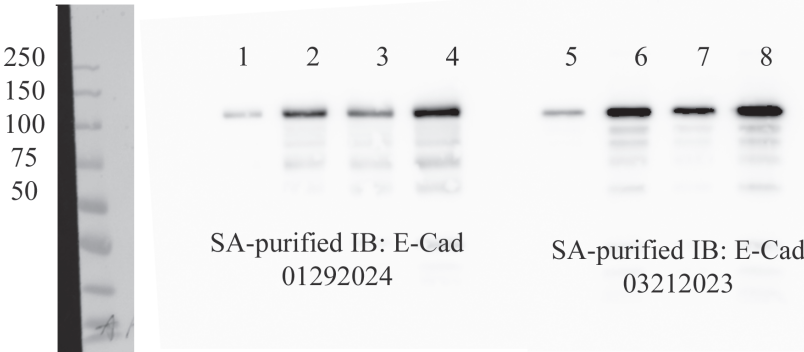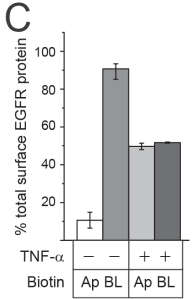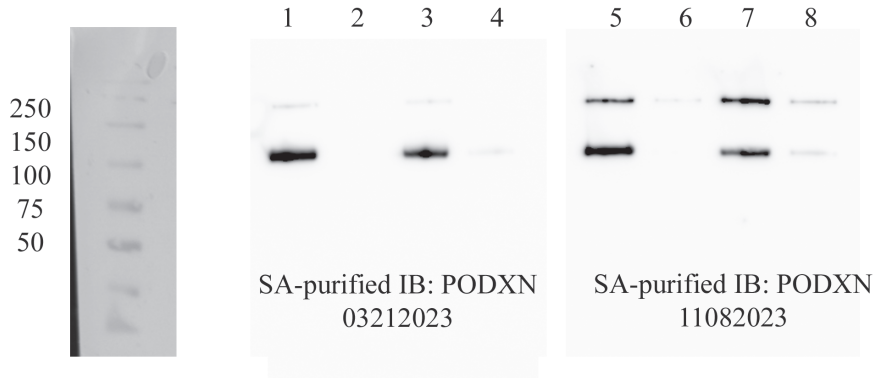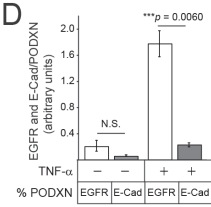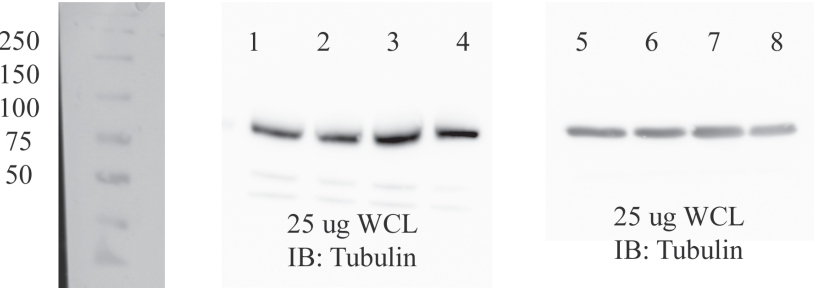

Supplement: Supplementary file 2 — Supplemental Figure S2. Raw data related to quantitative western blot analysis in Figure 2C,D. [file TRA-26-e70005-s007.pdf]

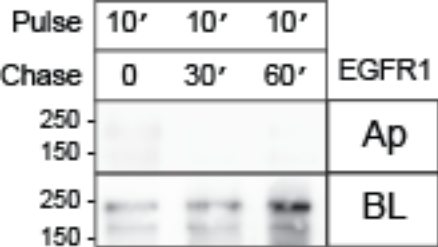

IB: TAMRA

Supplement: Supplementary file 5 — Supplemental Figure S5. Pulse‐chase analysis of domain‐specific EGFR delivery in untreated cells. [file TRA-26-e70005-s001.pdf]

Figure 5C

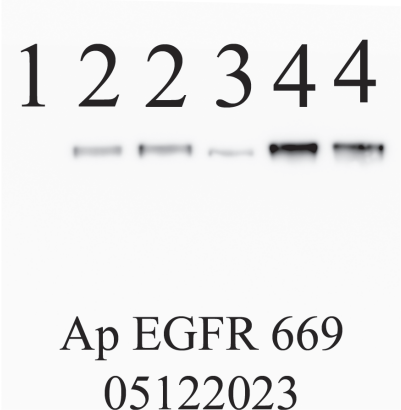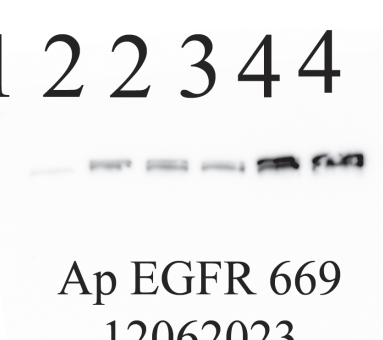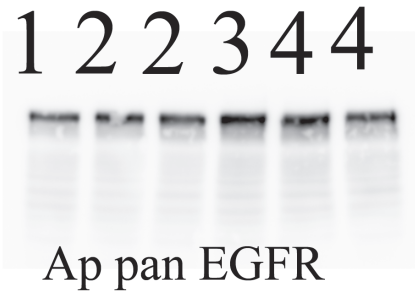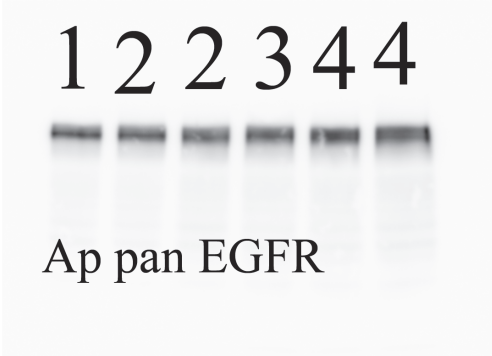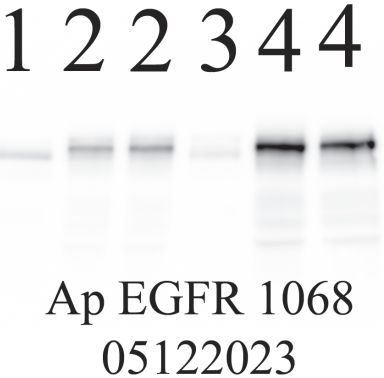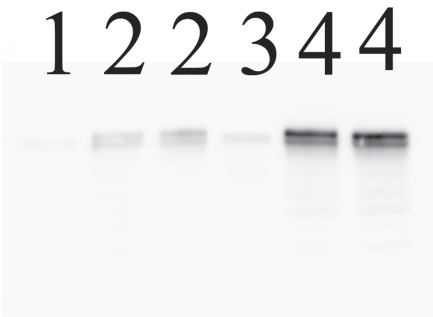

1 basal no egf  
2 basal EGF  
3 TNFa no EGF  
4 TNFa +EGF

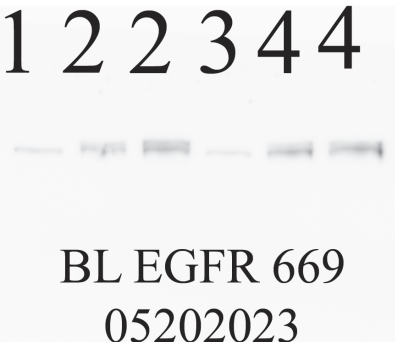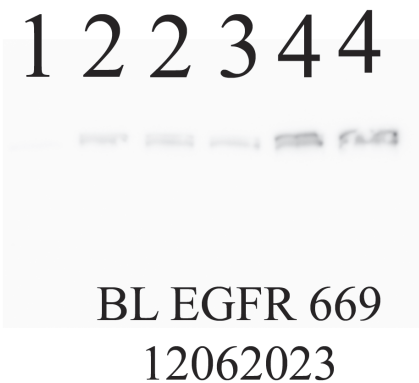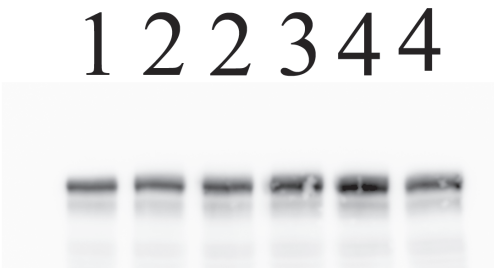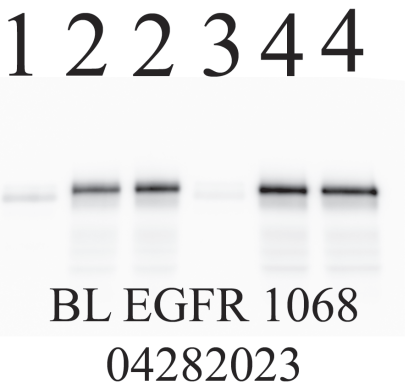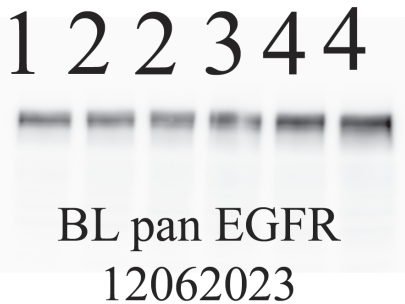

Supplement: Supplementary file 6 — Supplemental Figure S6. Raw data related to quantitative western blot analysis in Figure 5C. [file TRA-26-e70005-s002.pdf]

# Figure 5F

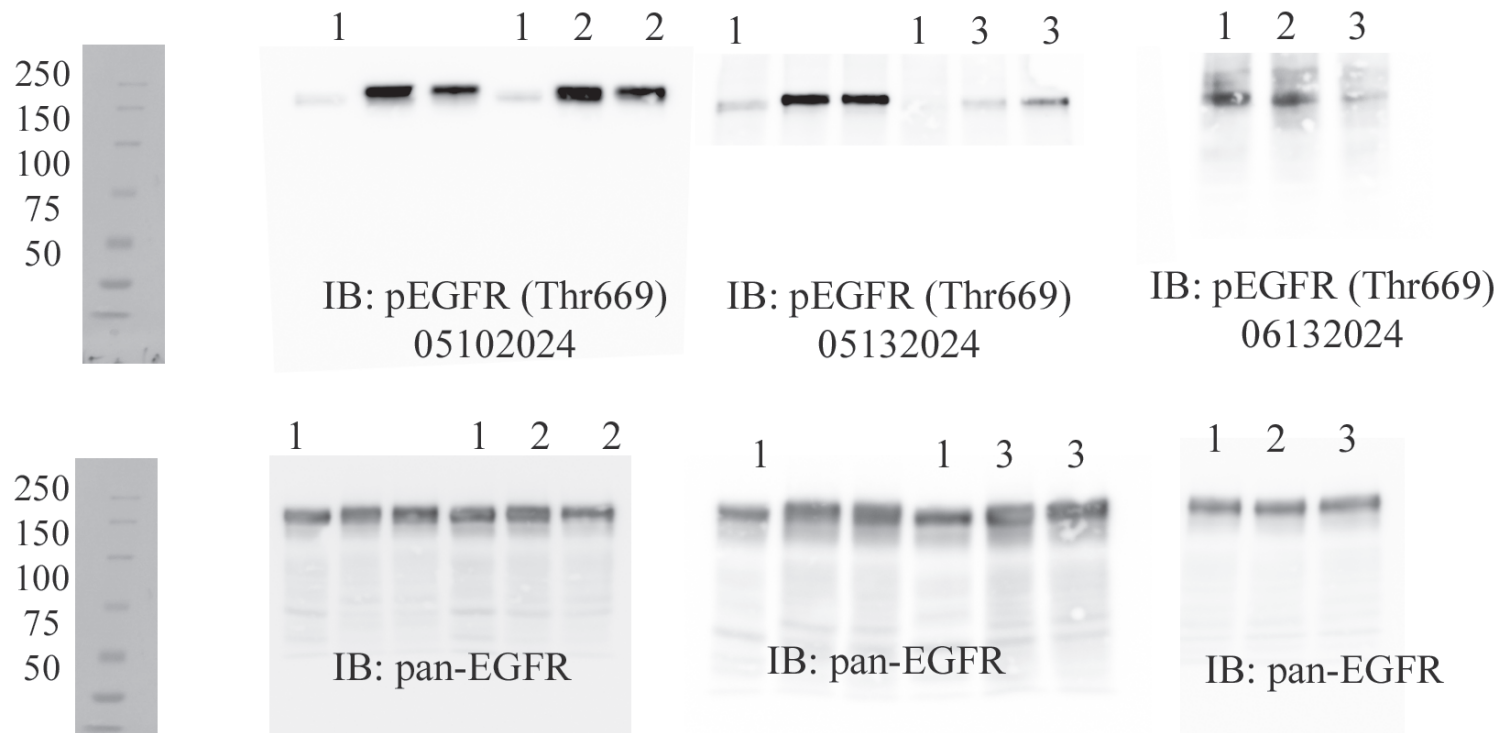

ALL: TNF-a, Ap EGF, 25 ug WCL  
 1 Vehicle  
 2 SB203580  
 3 U0126

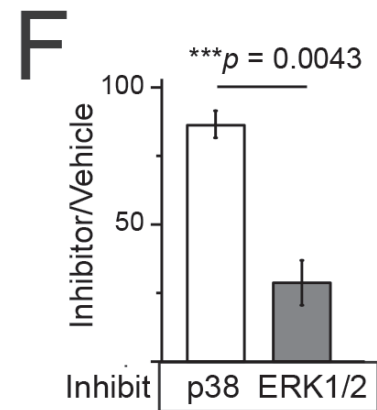

Supplement: Supplementary file 7 — Supplemental Figure S7. Raw data related to quantitative western blot analysis in Figure 5F. [file TRA-26-e70005-s009.pdf]

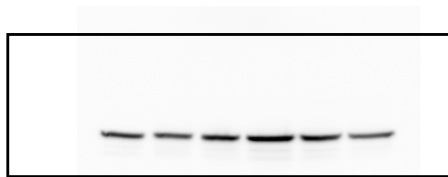

Figure 7B

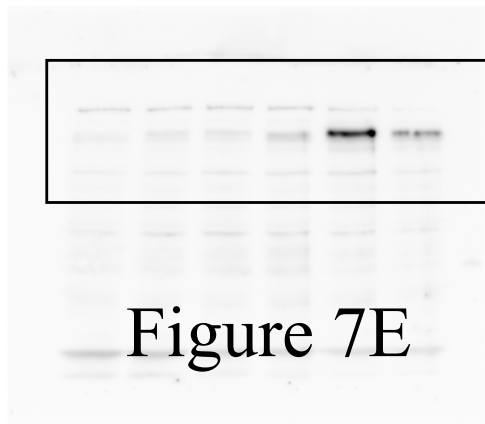

Figure 7E

Supplement: Supplementary file 10 — Data S1. Supporting Data. [file TRA-26-e70005-s006.pdf]
