# Supplementary material for: TNF‐α‐Driven Changes in Polarized EGF Receptor Trafficking Facilitate Phosphatidylinositol 3‐Kinase/Protein Kinase B Signaling From the Apical Surface of MDCK Epithelial Cells
Source: Traffic. 2025 May 5;26(4-6):e70005. doi: 10.1111/tra.70005 (PMC12052438; doi:10.1111/tra.70005)

Fig. 2F. Z-stack. TNFa (24 h). Stain for EGFR from Apical membrane non-permeabilized cells

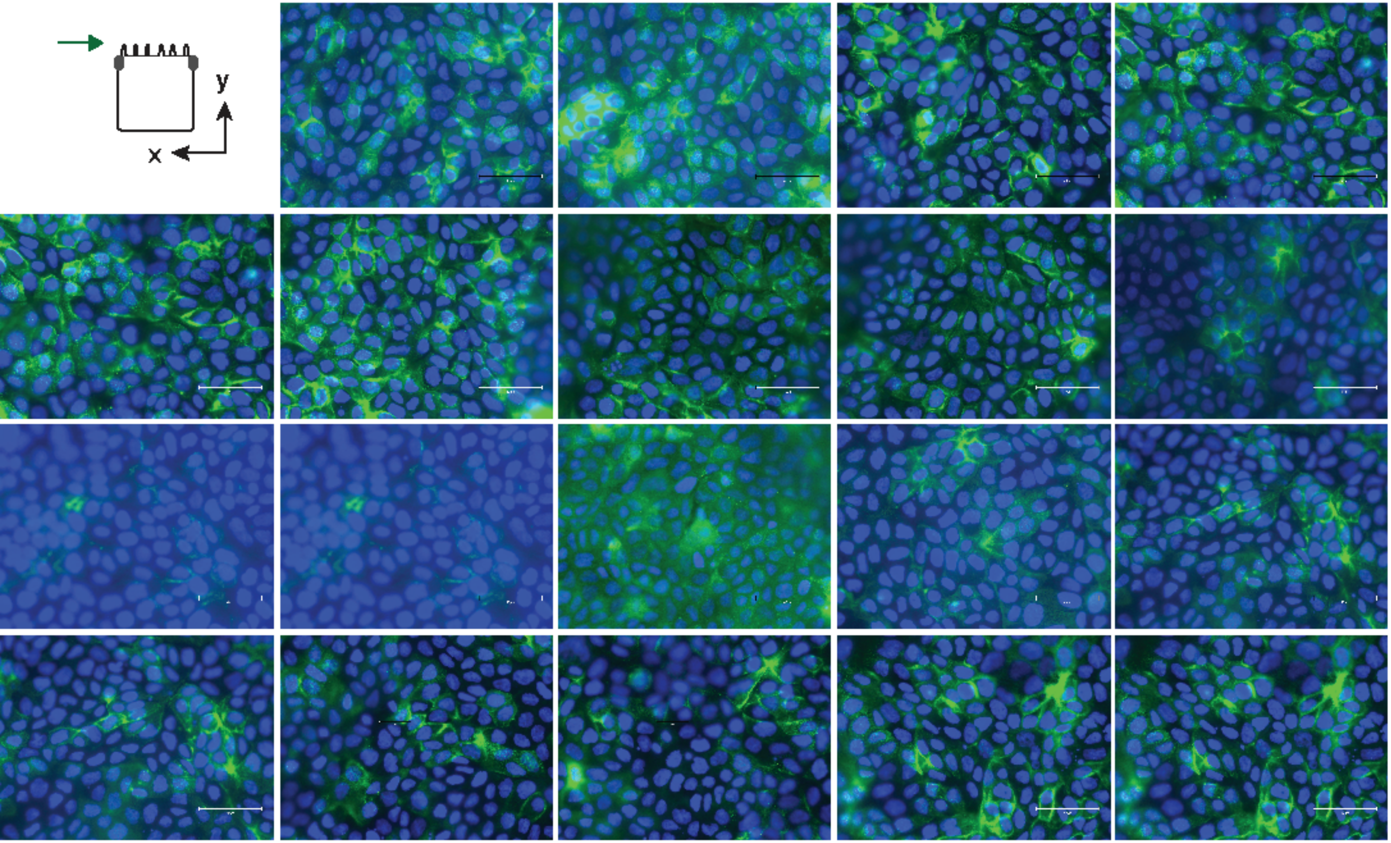

Fig. 2F. Z-stack. TNFa (24 h). Stain for EGFR from Basolateral membrane non-permeabilized cells

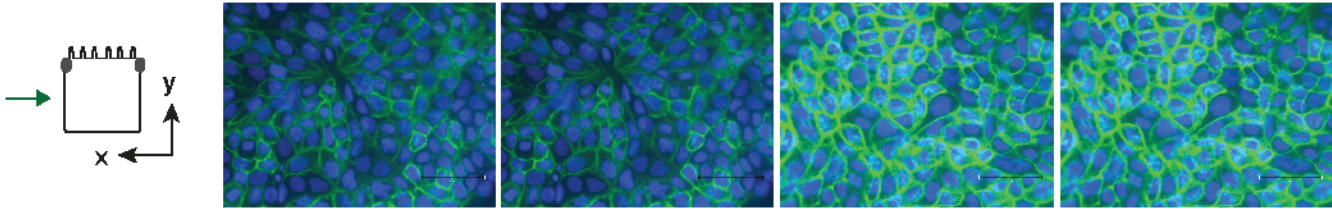

Supplement: Supplementary file 3 — Supplemental Figure S3. Raw Z‐stack images related to Figure 2F. [file TRA-26-e70005-s011.pdf]
