# Supplementary material for: TNF‐α‐Driven Changes in Polarized EGF Receptor Trafficking Facilitate Phosphatidylinositol 3‐Kinase/Protein Kinase B Signaling From the Apical Surface of MDCK Epithelial Cells
Source: Traffic. 2025 May 5;26(4-6):e70005. doi: 10.1111/tra.70005 (PMC12052438; doi:10.1111/tra.70005)
Supplement: Supplementary file 4 — Supplemental Figure S4. Raw data related to quantitative western blot analysis in Figure 4C,H. [file TRA-26-e70005-s005.pdf]

Figure 4C

All: 10 min pulse-label
0 minus EGFR1 control
1 No chase
2 30-min chase
3 60 min chase
4 90 min chase

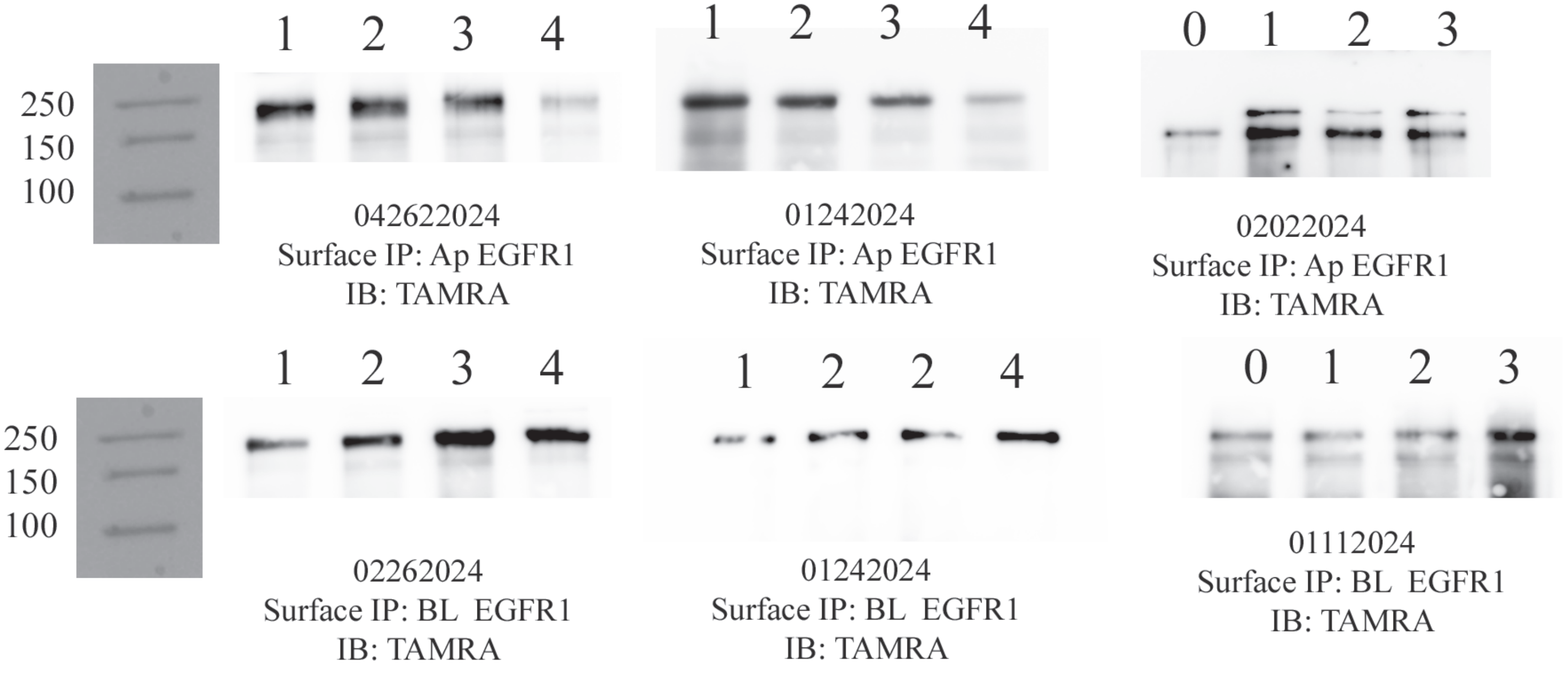

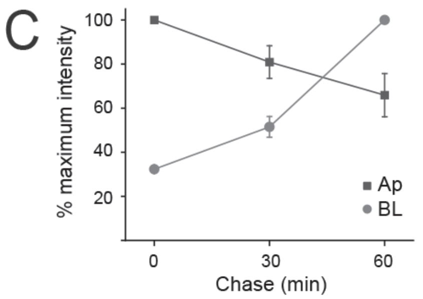

Figure 4H

1 37 degree C
2 20 degree
3 37 degree BFA

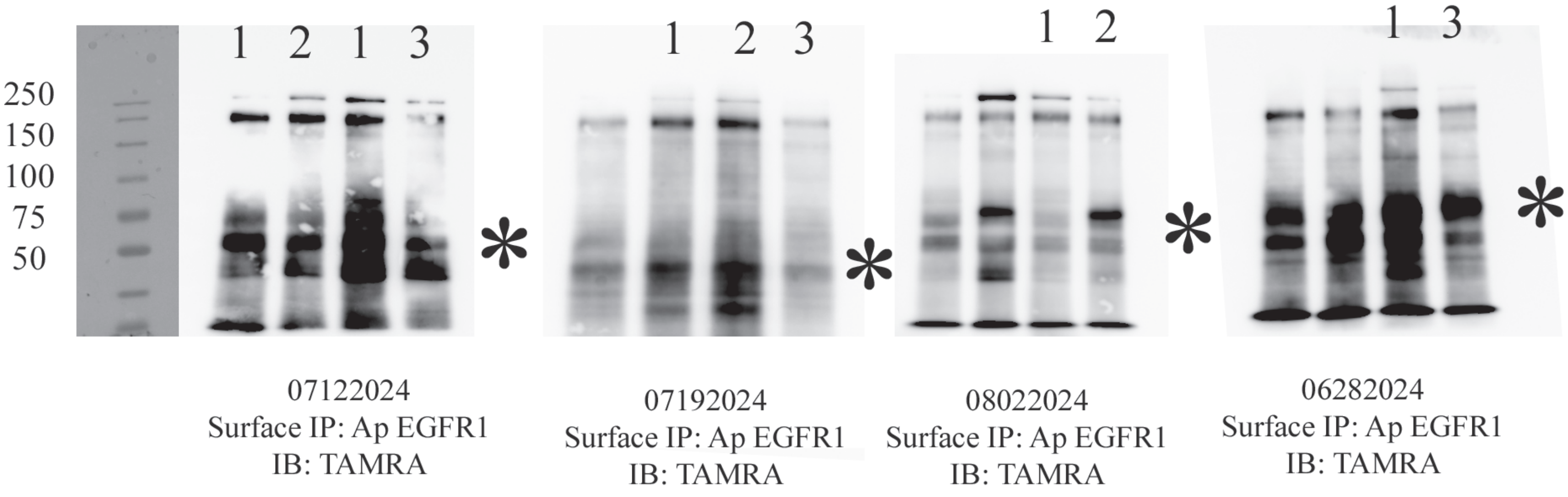

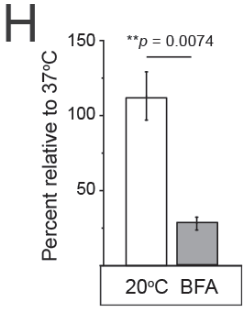

*IgG heavy chain from IP antibody
