# Supplementary material for: TNF‐α‐Driven Changes in Polarized EGF Receptor Trafficking Facilitate Phosphatidylinositol 3‐Kinase/Protein Kinase B Signaling From the Apical Surface of MDCK Epithelial Cells
Source: Traffic. 2025 May 5;26(4-6):e70005. doi: 10.1111/tra.70005 (PMC12052438; doi:10.1111/tra.70005)
Supplement: Supplementary file 8 — Supplemental Figure S8. Raw data related to quantitative western blot analysis in Figure 6B,C. [file TRA-26-e70005-s003.pdf]

Fig 6B

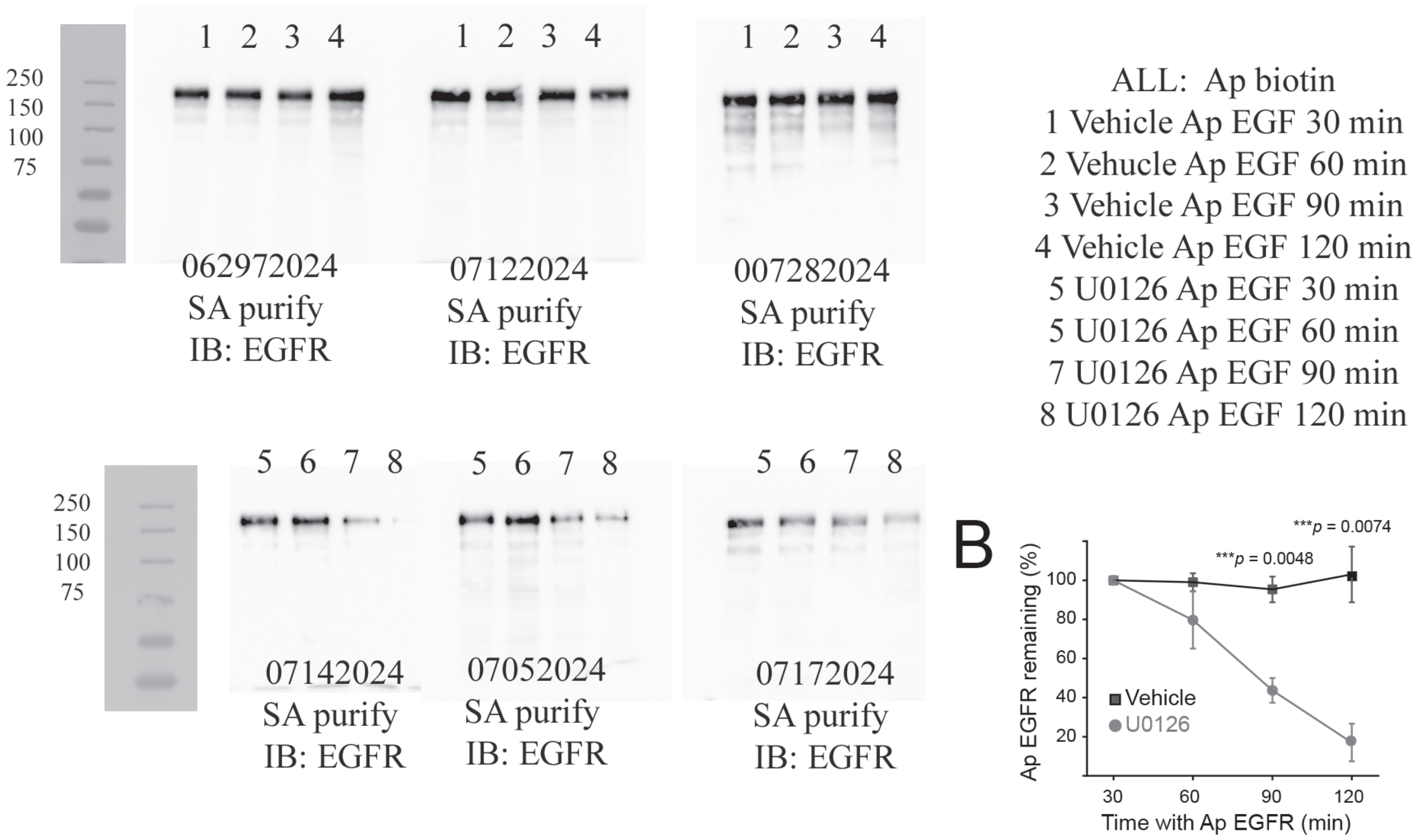

1 2 3 4
1 2 3 4
1 2 3 4
250
150
100
75
062972024
SA purify
IB: EGFR
07122024
SA purify
IB: EGFR
007282024
SA purify
IB: EGFR
ALL: Ap biotin
1 Vehicle Ap EGF 30 min
2 Vehucle Ap EGF 60 min
3 Vehicle Ap EGF 90 min
4 Vehicle Ap EGF 120 min
5 U0126 Ap EGF 30 min
5 U0126 Ap EGF 60 min
7 U0126 Ap EGF 90 min
8 U0126 Ap EGF 120 min
5 6 7 8
5 6 7 8
5 6 7 8
07142024
SA purify
IB: EGFR
07052024
SA purify
IB: EGFR
07172024
SA purify
IB: EGFR
B
***p = 0.0074
***p = 0.0048
Ap EGFR remaining (%)
Vehicle
U0126
Time with Ap EGFR (min)

Fig 6D

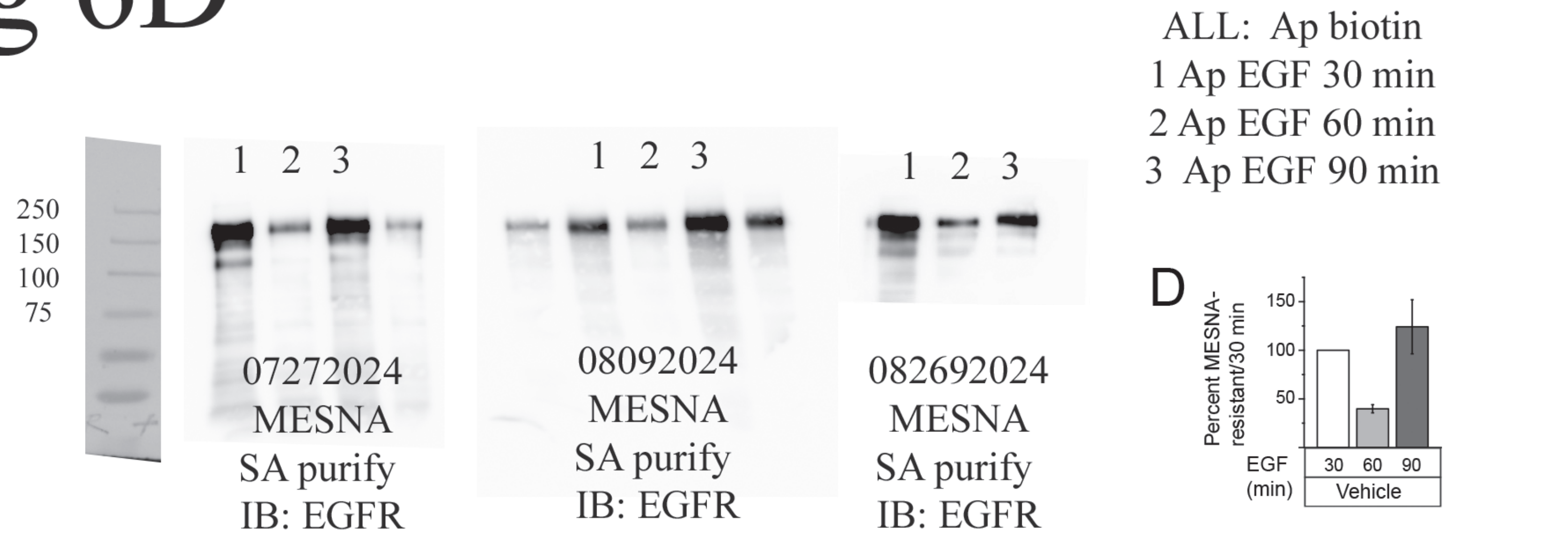

ALL: Ap biotin
1 Ap EGF 30 min
2 Ap EGF 60 min
3 Ap EGF 90 min
1 2 3
1 2 3
1 2 3
250
150
100
75
07272024
MESNA
SA purify
IB: EGFR
08092024
MESNA
SA purify
IB: EGFR
082692024
MESNA
SA purify
IB: EGFR
D
Percent MESNA-resistant/30 min
EGF (min)
30 60 90
Vehicle
