# Supplementary material for: TNF‐α‐Driven Changes in Polarized EGF Receptor Trafficking Facilitate Phosphatidylinositol 3‐Kinase/Protein Kinase B Signaling From the Apical Surface of MDCK Epithelial Cells
Source: Traffic. 2025 May 5;26(4-6):e70005. doi: 10.1111/tra.70005 (PMC12052438; doi:10.1111/tra.70005)

Fig. 7C

1 Basal no EGF
2 Basal Ap EGF 15 min
3 Basal Ap EGF 30 min
4 TNFa no EGF
5 TNFa Ap EGF 15 min
6 TNFa Ap EGF 30 min

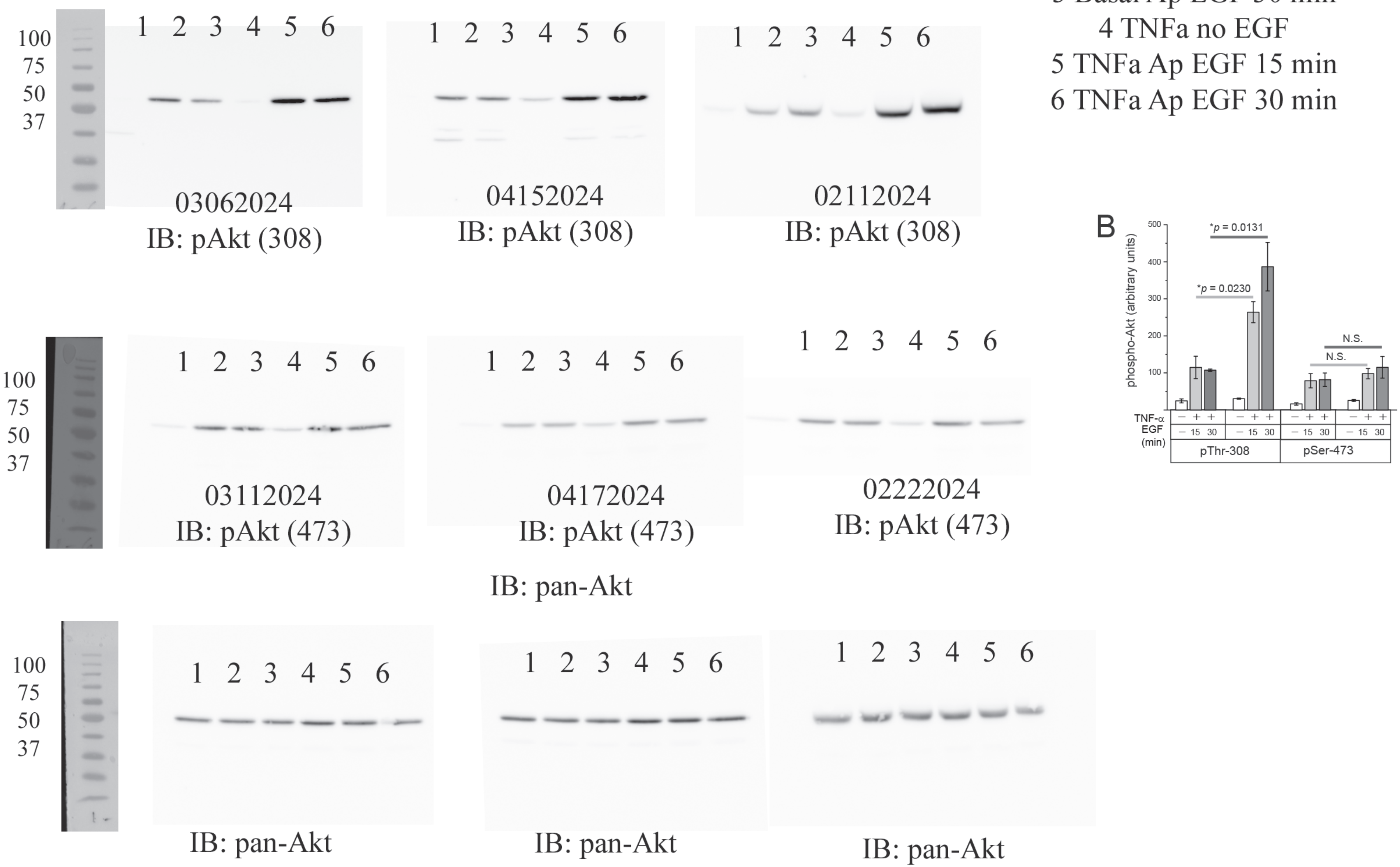

Supplement: Supplementary file 9 — Supplemental Figure S9. Raw data related to quantitative western blot analysis in Figure 7C. [file TRA-26-e70005-s010.pdf]
