# Supplementary material for: TNF‐α‐Driven Changes in Polarized EGF Receptor Trafficking Facilitate Phosphatidylinositol 3‐Kinase/Protein Kinase B Signaling From the Apical Surface of MDCK Epithelial Cells
Source: Traffic. 2025 May 5;26(4-6):e70005. doi: 10.1111/tra.70005 (PMC12052438; doi:10.1111/tra.70005)
Supplement: Supplementary file 11 — Supplementary Table 1. Comprehensive list of primary antibodies used in this study. [file TRA-26-e70005-s004.docx]

| **3000 MW** | | | | | | | |  | | | | | | | | |  |
| --- | --- | --- | --- | --- | --- | --- | --- | --- | --- | --- | --- | --- | --- | --- | --- | --- | --- |
| **3000 MW** | | | |  | | | | **40,000 MW** | | | | |  | | | |  |
| Basal | | TNF-α (24-h) | |  | |  | | Basal | | TNF-α (24-h) | | |  | |  | |  |
| μg* | %** | ug | % |  |  |  |  | μg | % | μg | % | |  |  |  | % |  |
| 1 | <0.040 | <0.16 | 0.184 | 0.736 |  |  |  |  | 0.037 | 0.074 | 0.211 | | 0.422 |  |  |  | 0.902 |
| 2 | <0.040 | <0.16 | 0.182 | 0.728 |  |  |  |  | 0.04 | 0.08 | 0.211 | | 0.455 |  |  |  | 0.89 |
| 3 | <0.040 | <0.16 | 0.181 | 0.724 |  |  |  |  | 0.037 | 0.074 | 0.214 | | 0.428 |  |  |  | 0.89 |
| Mean  ± sem | <0.040 | **<0.16** | 0.182  ±0.002 | 0.729  +0.004 |  |  |  |  | 0.038  ± 0.001 | **0.076**  **± 0.002** | 0.212  +0.001 | | 0.435  +0.010 |  |  |  | **0.894**  **± 0.004** |

* μg dextran in BL chamber 30 min × 37°C

** Percentage of total added to Ap chamber
